# Improved NDVI based proxy leaf-fall indicator to assess rainfall sensitivity of deciduousness in the central Indian forests through remote sensing

**DOI:** 10.1038/s41598-020-74563-2

**Published:** 2020-10-19

**Authors:** Beependra Singh, C. Jeganathan, V. S. Rathore

**Affiliations:** Department of Remote Sensing, Birla Institute of Technology (BIT), Mesra, Ranchi, Jharkhand 835215 India

**Keywords:** Climate sciences, Ecology, Environmental sciences, Environmental social sciences

## Abstract

Quantifying the leaf-fall dynamics in the tropical deciduous forest will help in modeling regional energy balance and nutrient recycle pattern, but the traditional ground-based leaf-fall enumeration is a tedious and geographically limited approach. Therefore, there is a need for a reliable spatial proxy leaf-fall (i.e., deciduousness) indicator. In this context, this study attempted to improve the existing deciduousness metric using time-series NDVI data (MOD13Q1; 250 m; 16 days interval) and investigated its spatio-temporal variability and sensitivity to rainfall anomalies across the central Indian tropical forest over 18 years (2001–2018). The study also analysed the magnitude of deciduousness during extreme (i.e., dry and wet) and normal rainfall years, and compared its variability with the old metric. The improved NDVI based deciduousness metric performed satisfactorily, as its observed variations were in tandem with ground observations in different forest types, and for different pheno-classes. This is the first kind of study in India revealing the spatio-temporal character of leaf-fall in different ecoregions, elevation gradients and vegetation fraction.

## Introduction

Tropical forests are well known for their role in global carbon cycle^1,2^, biomass accumulation^3^, regulating climatic services^4,5^, water use in different rooting pattern at different depths^6,7^ and influencing regional climate including precipitation distribution^8^. The recent abrupt changes in the intensity, frequency and distribution of world's climatic extreme events (drought, heat-wave, forest fires and floods) have adversely impacted the structure and functioning of tropical ecosystems^9–11^. However, deciduous forests of tropical regions are well known for their quick adaptability to xeric conditions and exhibit periodic phenomena like flowering, fruiting, vegetative growth, and leaf-fall at different times of the year which varies with places as per their adaptation to local weather conditions^12^. Although, most trees shed their leaves all through the year, their leave shedding ability (called deciduousness) varies across seasons, but maximum leaf-fall occurs during the dry season. Therefore, deciduousness of tropical forests is considered to be one of the important indicators of climate change due to its strong dependency on rainfall and temperature^13,14^. Thus, understanding the spatio-temporal pattern of deciduousness is useful in predicting carbon dynamics, water use efficiency, nutrient cycle and litter fall of the forest floor. Though many studies^15–17^ have been carried out on vegetation phenology using the traditional ground-based techniques and satellite data, observing a significant change in the phenological behaviour requires a long time observation. However, the intra-annual variations in the weather pattern and its immediate link on the vegetation growth still need further recurring evidence. Importantly, the Central Indian Landscape is highly drought-prone, and tropical deciduous forests have unique ability to quickly adapt to adverse conditions. In this regard, it would be interesting to study the spatial pattern of leaf-fall dynamics in the vast regions of tropical deciduous forests of the central India in different rainfall conditions.

Globally, few studies have been carried out in quantifying deciduousness from remote sensing data. Cuba et al.^14^ studied the spatial pattern of deciduousness over 11 years in Mexican Yucatan forest using Enhanced Vegetation Index (EVI) based metric. We used Normalised Difference Vegetation Index (NDVI) instead of EVI in this study as the NDVI from MODIS and GIMMS has been extensively used for vegetation monitoring^15,18–21^. Also, the recent study on phenology by Rankine et al.^22^ in a tropical dry forest observed greater strength of in-situ and MODIS NDVI relationship than EVI.

Gandolfi et al.^23^ discussed the significance of modeling the leaf-fall as it affects the regenerations, growth and helps in maintaining the biodiversity in the under-storey micro-habitat in the long run. Increasing frequency of extreme weather events would affect the photosynthesis, leaf-fall pattern, and hence would alter adaptations and survival strategies of tropical forest under stress conditions. Several studies have reported 10 to 30% of increase in extreme events (e.g., severe droughts of 2002, 2009, 2014 and 2015) over the Central India^24–27^ which led to increase in forest fires in the dry deciduous forests^28–30^. Moreover, these extreme events will most probably also impact plants natural process of germinations, regeneration and mortality and the entire process of ecological succession in the region. So, it is important to analyze the past and present vegetation conditions, and its resilience and vulnerability towards future climate change scenarios. It is believed that the Central Indian deciduous forest plays an important localized role in controlling monsoon. Thus, any impact on the existence of this forest will probably impact the rainfed agriculture in this region. Evergreen, semi-evergreen, dry deciduous and moist deciduous forests are the major types of forests of the Indian subcontinent where the tropical forest covers maximum land surface area (approximately 69%)^31^.

**Figure 1:**
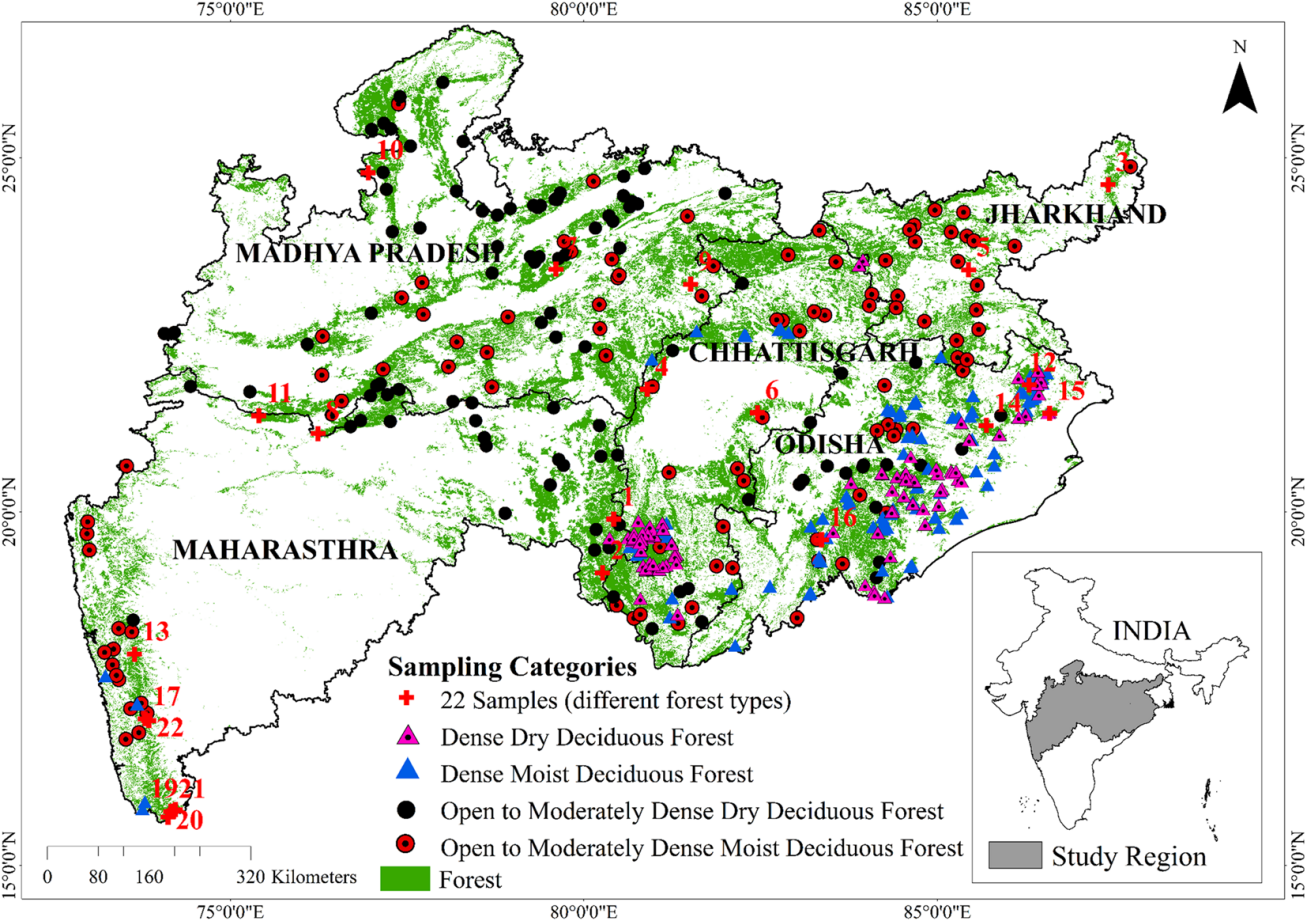
Study area showing distribution of forests with locations of samples from different forest types. (This map was created using ESRI's ArcMap 10.3—https://desktop.arcgis.com/en/arcmap/, and MS-Office PowerPoint 2007 software).

The Central Indian deciduous landscape provides shelter for different flora and fauna including the tiger, the important and endangered animal^32^. In addition, it supports huge population for their livelihood, fuel, biomass, medicine, clean water, forest products and other ecological services. This region is also a source of Tendu leaf—*Diospyros melanoxylon* which is used for manufacturing the "Bidi" (i.e. cigarettes). Millions of the local community in Madhya Pradesh, Odisha and Chhattisgarh are involved in collecting Tendu leaf and non-timber forest products (*Madhuca indica*) for their livelihood^33–35^. Though, many isolated ground-level studies were carried out to understand the species-level growth pattern^12,36,37^, it is important to know the collective behaviour of a forest at a landscape level rather than the species specific for understanding the regional pattern (Fig. 1)^38^.

In this context, the present study aims to (1) develop a remote sensing based metric which is sensitive and efficient to quantify deciduousness, (2) characterize the deciduousness pattern in the Central India and analyse its spatio-temporal variability and (3) examine the sensitiveness of deciduousness to extreme rainfall conditions.

## Results

### Comparison between old and new deciduousness metrics

**Figure 2:**
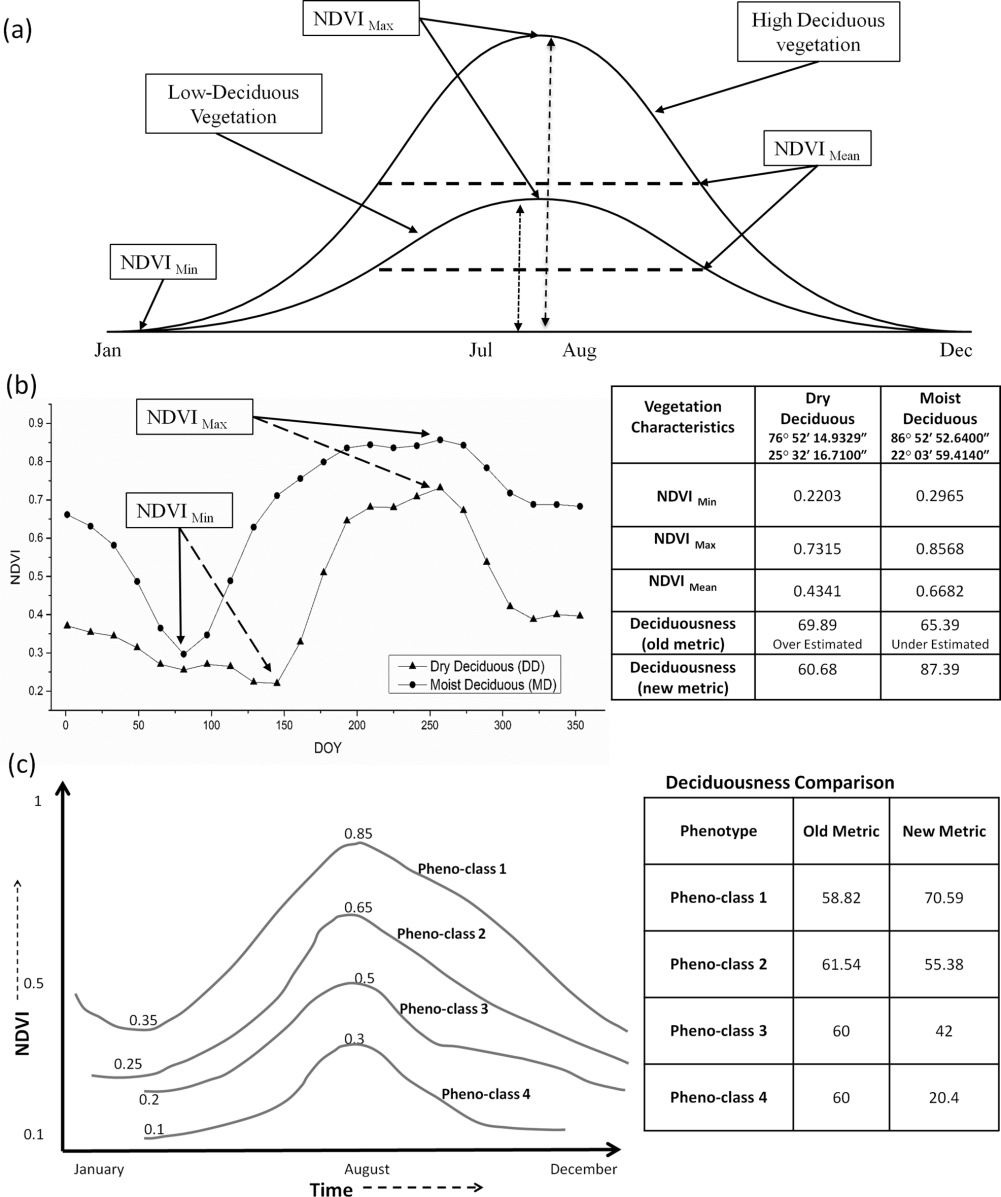
Graphical illustration of deciduousness estimation: (**a**) Theoretical phenology curves from high and low deciduous vegetation and the parameters of deciduousness, (**b**) Actual RS derived annual growth profiles of moist and dry deciduous vegetation and their deciduousness estimation using the old and new metric, and (**c**) Annual growth profile of four theoretical pheno-classes for depicting the different magnitude of deciduousness.

At first, to check the reliability of the proposed metric, we estimated the deciduousness from the equation proposed by Cuba et al.^14^ (Eq. 1; referred as ‘old’) and the new metric proposed in this study (Eq. 2; referred as ‘new’) during the extreme and normal rainfall years. The results of dry and moist deciduous samples and 4 pheno-classes revealed an over-estimation and under-estimation of deciduousness with the old-metric, whereas the new metric revealed the accurate relative variability (Fig. 2b,c, Table S1). Table 1 provides the estimated deciduousness values from the old and new metrics for 22 homogeneous sample pixels representing four major vegetation types in the study area (refer Fig. 1 for their spatial locations and Fig. S1 for their annual growth profile). The litter fall information collected from literature revealed a higher litter fall quantity of 10–14.4 Mg Ha^−1^ year^−1^ for the moist deciduous forest^39–42^ and lower litter fall quantities of 1–8.65 Mg Ha^−1^ year^−1^ and 5.63–7.84 Mg Ha^−1^ year^−1^ for the dry deciduous forest^42–44^ and the semi-evergreen and evergreen forest^44^ , respectively. The new metric showed a relatively similar variability in deciduousness to ground observations especially for the moist and dry-deciduous forests than the old metric (Table 1).

**Table 1:** Performance of old and new deciduous metric in a normal rainfall year (2011) using 22 samples from different vegetation types (spatial locations of these samples can be seen in Fig. 1).

Sample ID	Forest types	NDVI _Min_	NDVI _Max_	NDVI _Mean_	Old metric	New metric
S1	Moist deciduous (MD)	0.4438	0.8555	0.6668	48.13	64.18
S2		0.5126	0.9211	0.7515	44.35	66.66
S3		0.5247	0.8587	0.7252	38.90	56.42
S4		0.3989	0.8510	0.6054	53.12	64.31
S5		0.4010	0.7779	0.5942	48.44	57.57
S6		0.4346	0.8421	0.6845	48.39	66.25
S7	Dry deciduous (DD)	0.2645	0.6636	0.4141	60.14	49.80
S8		0.2390	0.6656	0.4082	64.09	52.32
S9		0.2585	0.7127	0.4322	63.73	55.09
S10		0.1907	0.6631	0.3604	71.24	51.34
S11		0.2592	0.5640	0.4034	54.04	43.60
S12	Semi-evergreen (SE)	0.7198	0.8871	0.8045	18.86	30.35
S13		0.7423	0.8379	0.8020	11.42	18.31
S14		0.7226	0.8730	0.8133	17.23	28.02
S15		0.7886	0.9094	0.8494	13.28	22.56
S16		0.7400	0.8845	0.8212	16.34	26.84
S17		0.7022	0.8564	0.7819	18.01	28.16
S18	Evergreen (E)	0.8033	0.8434	0.8180	4.75	7.78
S19		0.7959	0.8551	0.8213	6.92	11.37
S20		0.7615	0.8446	0.8091	9.83	15.91
S21		0.8002	0.8645	0.8244	7.44	12.26
S22		0.7928	0.8398	0.8167	5.60	9.15

**Figure 3:**
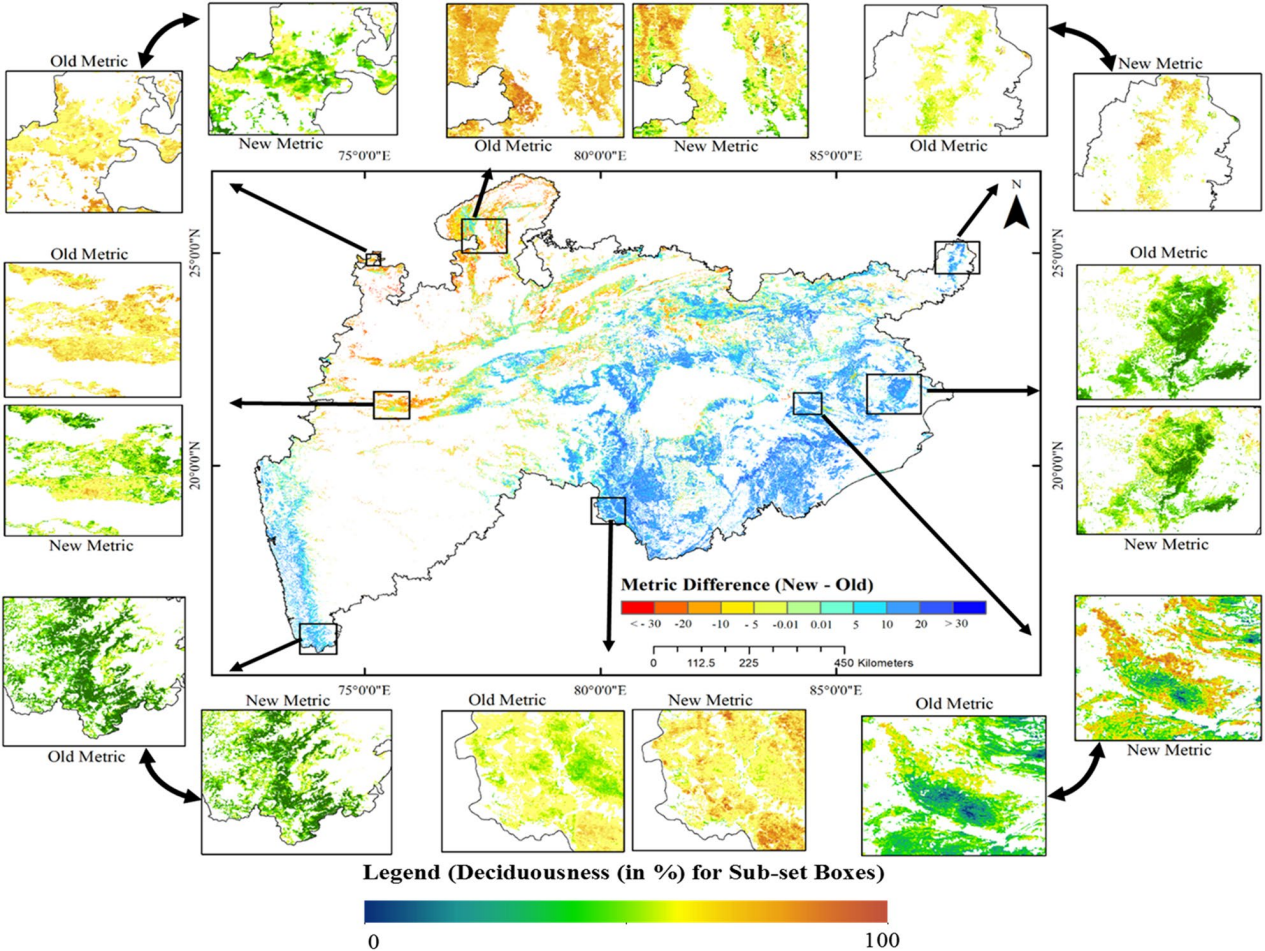
Difference in the spatial distribution of deciduousness (central figure) and the actual deciduousness (subset boxes) derived from the new and old metric for the year 2011. (These maps were created using ESRI's ArcMap 10.3—https://desktop.arcgis.com/en/arcmap/, and MS-Office PowerPoint 2007 software).

Further, the difference between the old and new metric was spatially checked and is shown at the center of Fig. 3, and the actual values are presented in the surrounding in eight different sub-set locations. The difference image denotes the under-estimated (70.76% of forest area) and the over-estimated (29.23% of forest area) deciduousness obtained by the old metric (Fig. 3). The under-estimated area observed was mainly in the moist forested regions of states- Chhattisgarh, Odisha, and Jharkhand states, whereas, the over-estimated area observed was mainly in the dry forested region of states—Madhya Pradesh, Maharashtra, Northern Chhattisgarh and some parts of Jharkhand (Fig. 3). The over- and under-estimations are with respect to the new metric, and not with the real in-situ measurements. However, the new metric is in good agreement with annual growth profiles of different vegetation types, and have positive relation with ground litter fall observations^39–44^.

The deciduousness derived from these two metrics were also tested for their statistical significance using ANOVA (Table 2). In this test 800 stratified random samples belonging to different deciduous forests of different density classes for dry (2002), normal (2011) and wet (2013) years were used. It was found that the mean deciduousness values from the old metric were similar in the majority of the cases and different rainfall conditions. Hence, it could not be used for understanding rainfall impact on the deciduousness. On the other hand, the new metric performed better than the old metric in terms of its variability under (a) different rainfall conditions (p < 0.001), (b) different vegetation types (p < 0.001) and (c) different forest densities (p < 0.05). Thus, we used the new metric for further analysis to get a greater detail about the deciduousness behaviour of the Central Indian forests.

**Table 2:** Testing the performance of the old and new metric using ANOVA at different stratification levels.

	Summary	Dry deciduous			Moist deciduous		
New metric < 30% VCF (open to moderately dense forest)	Year	2002	2011	2013	2002	2011	2013
	Average	57.56	60.96	64.52	56.50	59.08	62.28
	SD	10.90	8.78	9.00	9.40	10.08	9.65
	Variance	118.83	77.05	80.94	88.35	101.53	93.17
	Standard error	1.09	0.88	0.90	0.94	1.01	0.97
	F	13.13	(Significant)		8.89	(Significant)	
	F critical	3.03			3.03		
	P value	0.000002		N = 100	0.000179		N = 100
Old metric < 30% VCF (open to moderately dense forest)	Average	61.71	61.01	62.80	51.62	52.52	52.58
	SD	7.79	9.71	8.50	10.21	11.12	10.73
	Variance	60.62	94.19	72.19	104.32	123.66	115.08
	Standard error	0.78	0.97	0.85	1.02	1.11	1.07
	F	1.07	(Not significant)		0.25	(Not significant)	
	F critical	3.03			3.03		
	P value	0.342711		N = 100	0.775487		N = 100
New metric > 40% VCF (dense forest)	Average	55.57	52.84	56.06	49.30	46.93	51.05
	SD	9.46	10.47	9.95	11.90	11.39	12.15
	Variance	89.42	109.52	98.92	141.59	129.76	147.51
	Standard error	0.96	1.06	1.00	1.21	1.16	1.23
	F	2.98	(Significant)		2.98	(Significant)	
	F critical	3.03			3.03		
	P value	0.052340		N = 98	0.052580		N = 97
Old metric > 40% VCF (dense forest)	Average	38.75	35.87	38.46	34.22	32.11	34.37
	SD	7.51	7.69	7.71	9.12	8.57	8.83
	Variance	56.47	59.10	59.45	83.17	73.41	78.03
	Standard error	0.75	0.77	0.77	0.93	0.87	0.90
	F	4.23	(Significant)		1.98	(Not significant)	
	F critical	3.03			3.03		
	P value	0.015516		N = 98	0.139391		N = 97

***SD standard deviation, *N* total number of samples.

### Spatio-temporal distribution and variation in deciduousness

**Figure 4:**
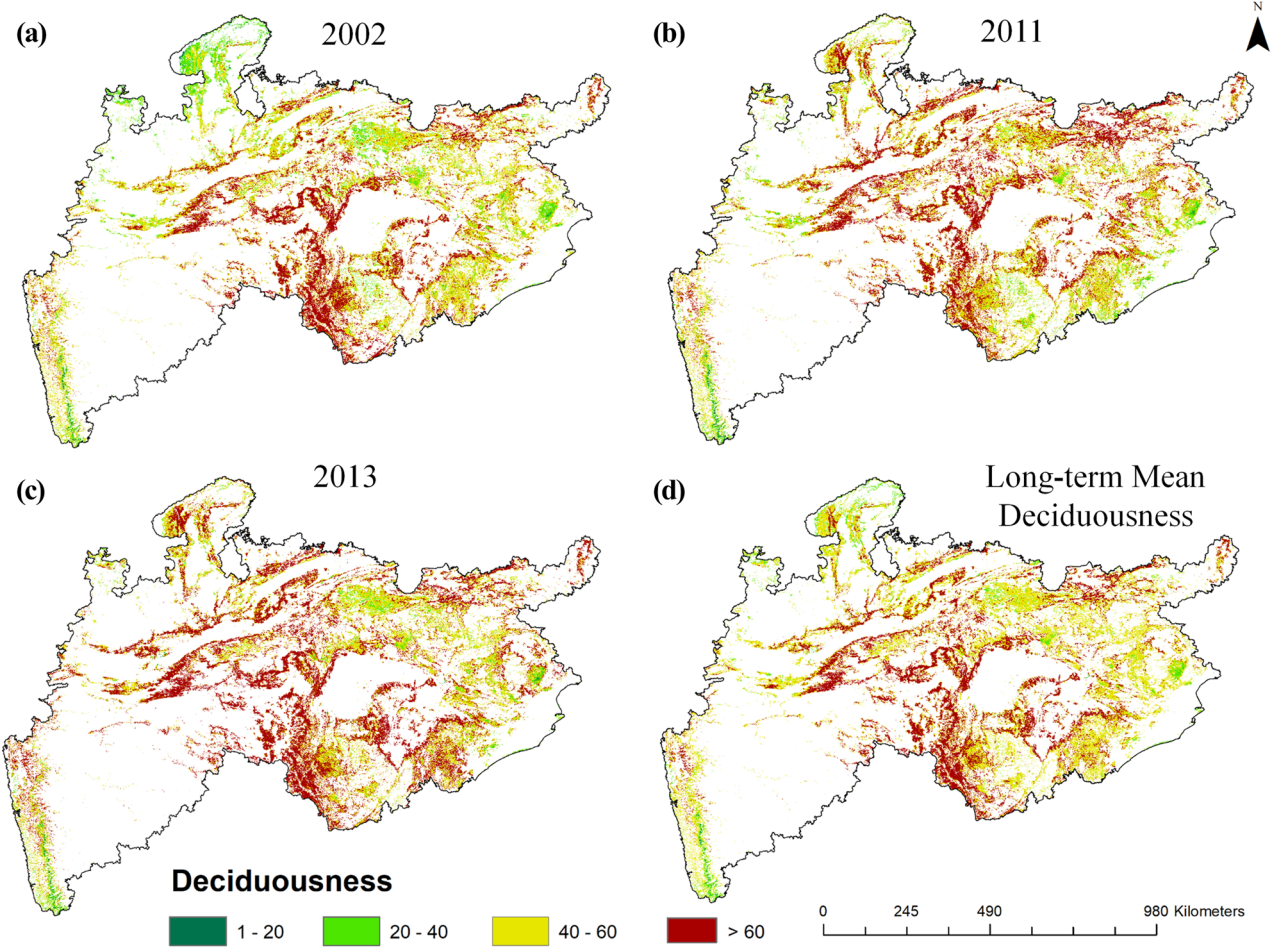
Spatial variability of deciduousness in dry year (2002) (**a**), normal year (2011) (**b**), wet year (2013) (**c**) and long-term mean deciduousness (**d**). (These maps were created using ESRI's ArcMap 10.3—https://desktop.arcgis.com/en/arcmap/, and MS-Office PowerPoint 2007 software).

We estimated Annual Deciduousness (AD) and Relative Annual Deciduousness (RAD) from 18 years (2001–2018) of MODIS NDVI data, and analysed their spatio-temporal variability in different rainfall scenarios i.e. during dry, normal and wet years (Fig. 4). For description purpose, we grouped deciduousness into four different classes such as: (1) 0–20% as low deciduousness (LD), (2) 20–40% as moderate deciduousness (MD), (3) 40–60% as high deciduousness (HD) and (4) above 60% as very high deciduousness (VHD). The intra- and inter-annual differences in spatial distribution and magnitude of deciduousness could be attributed to the effect of local, micro to macro-climatic regimes in the landscape.

From the rainfall anomaly pattern of 18 years (Fig. S2), it was observed that the year 2002 received the lowest rainfall, as 98.97% of the area was under negative anomaly and 47% of the area was under severe dry condition i.e., rainfall was much less than (< − 20%) the normal range. The year 2013 received the highest rainfall, where 45% of the area was under heavy rainfall (> + 20% of normal rainfall), and in the year 2011 more than 95% of the area received normal rainfall. So, these three years (2002, 2011 and 2013) were chosen as the representative years of extreme and normal conditions for further analysis of the deciduousness. During the dry spell around 12% of the study area experienced lower magnitude of deciduousness (Fig. 4a), especially in the ecoregions like Kathiar-Gir dry deciduous forests, Upper Gangetic plains, north-eastern parts of the Narmada valley, western parts of the Chhota-Nagpur and eastern parts of Eastern highlands. The hotspot pattern of VHD was observed only in the densely forested areas. In the year 2013 (wet year), healthy deciduousness was seen all over the study area with prominence in the Central Deccan Plateau (CDP) (Fig. 4c). A lower magnitude of deciduousness (~ 5%) was observed mainly in the non-deciduous forest types such as evergreen and semi-evergreen.

In the year 2011 (normal year), patches of VHD were observed in the CDP and some parts of the Chhota-Nagpur Region (Fig. 4b). Based on the long-term (18 years) mean deciduousness analysis around 36.85% of the forested area exhibited VHD (Fig. 4d), which was distributed as 11.5%, 8.82%, 8.13%, 5.16% and 3.24%, in states- Madhya Pradesh, Maharashtra, Chhattisgarh, Odisha and Jharkhand, respectively. The spatial distribution of long-term deciduousness (Fig. 4d) revealed a dominant deciduous nature of vegetation in this region. However, the high values of VHD were observed in the central-southern part of the study area and the low values in the evergreen and semi-evergreen forests of the Western Ghats and Similipal area of the Odisha state (Fig. 4d).

**Figure 5:**
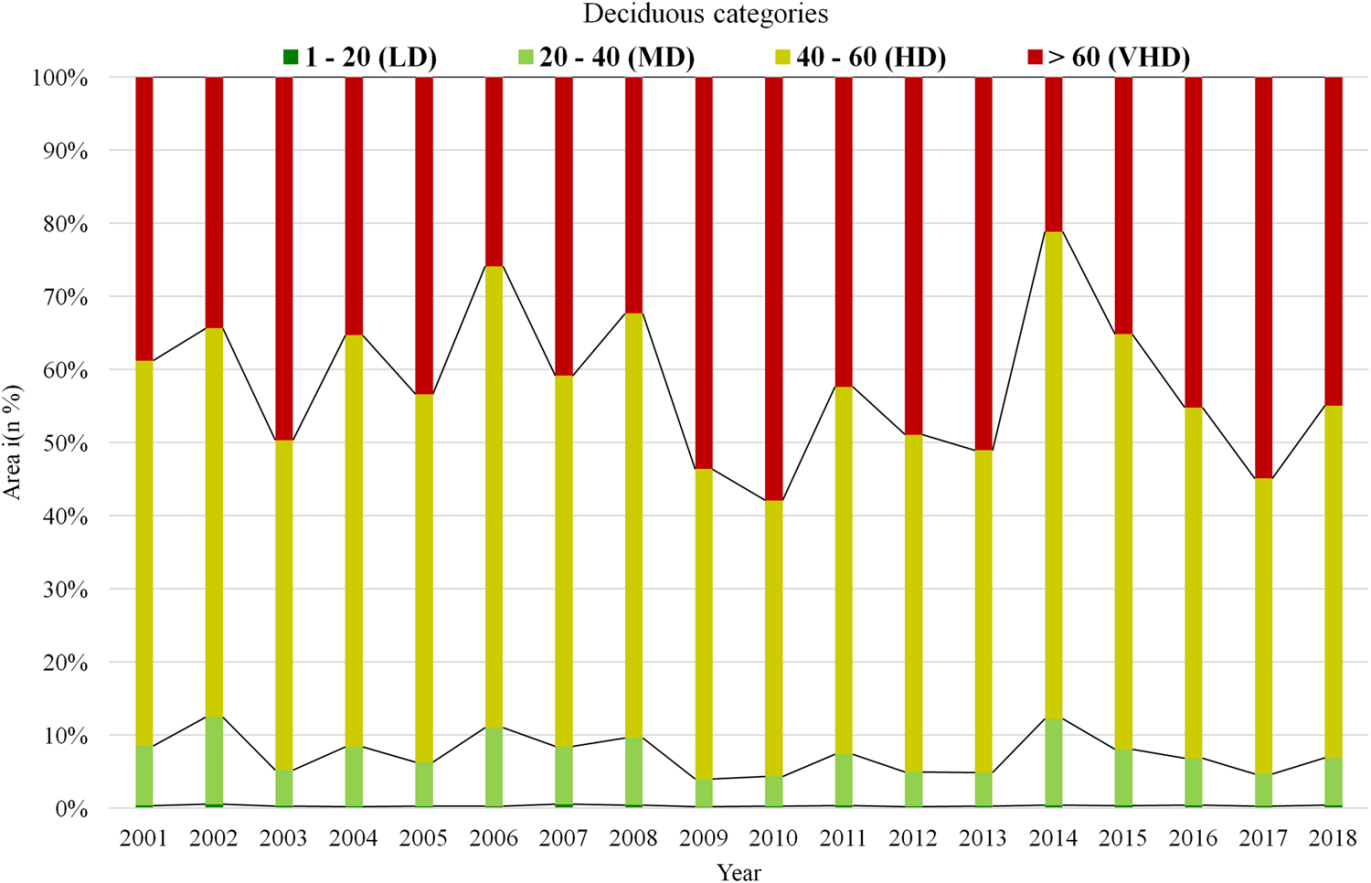
Temporal variation in the percentage area of different categories of deciduousness.

The yearly deciduousness images were classified into different categories and the variations observed during extreme years are listed in Table 3. The area statistics of deciduousness for all 18 years is provided in the supplementary data (Table S2). During the dry year, the forest area under VHD category was about 34.38% but in the normal year, it increased to 42.38%. In the wet year, it was the highest (51.06%) under VHD category**.** The area of deciduousness variability from 2001 to 2018 is presented in Fig. 5. During the study period (18 years), the mean deciduousness was observed to be fluctuating. A clear area reduction in VHD category during the dry years (i.e., 2002 and 2014), and increase during the wet years (2010 and 2017) was observed.

**Table 3:** Deciduousness distribution (in % area) during dry, normal and wet years.

Deciduousness classes	Deciduousness value range	Dry year (2002)	Normal year (2011)	Wet year (2013)	Long-term mean
Low deciduousness (LD)	(1–20)	0.53	0.38	0.29	0.19
Moderate deciduousness (MD)	(20–40)	11.94	7.00	4.56	5.12
High deciduousness (HD)	(40–60)	53.13	50.23	44.07	57.84
Very high deciduousness (VHD)	(> 60)	34.38	42.38	51.06	36.85

### Spatio-temporal distribution and variation in relative deciduousness (RAD)

**Figure 6:**
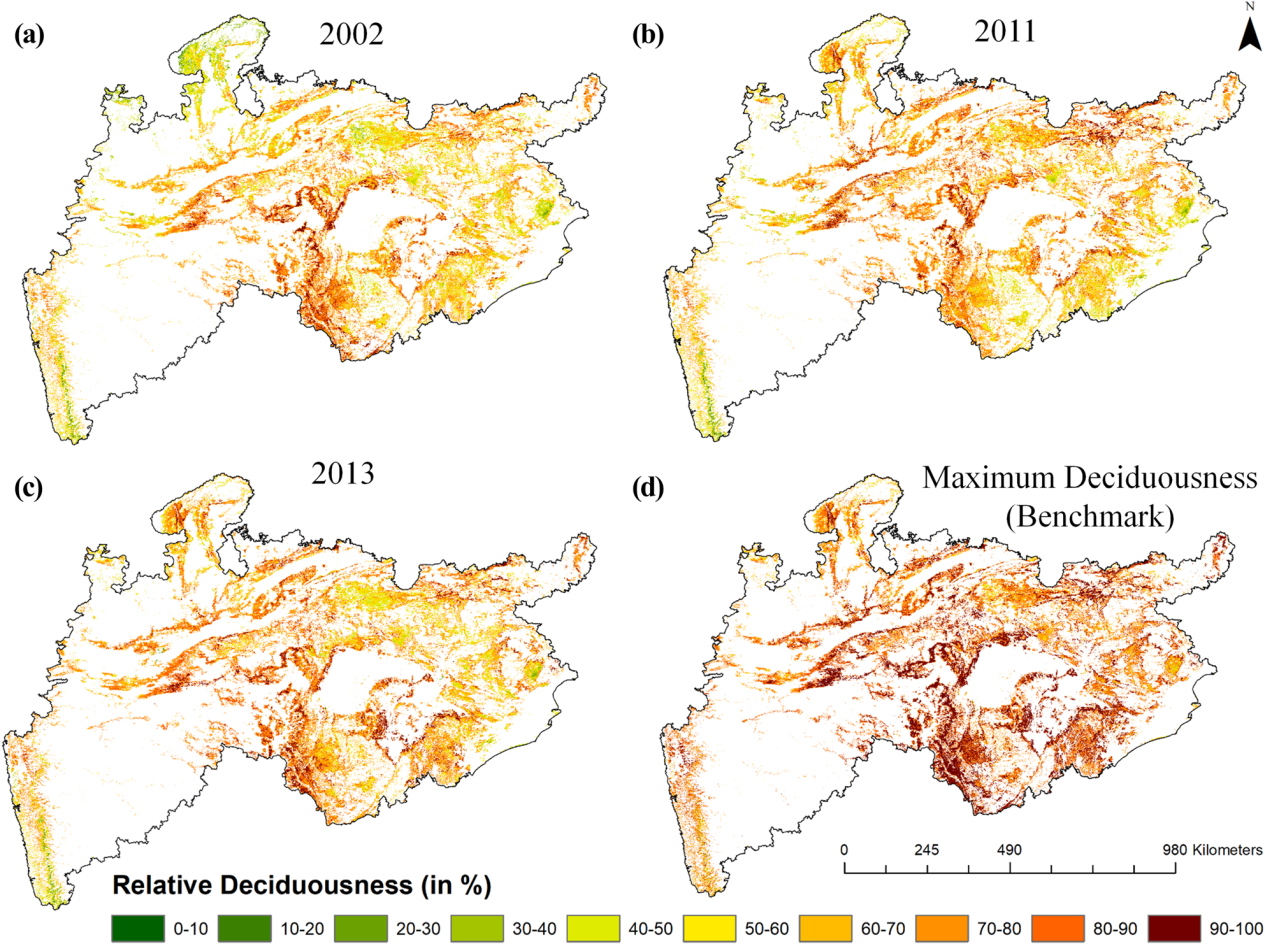
Spatial variability of relative deciduousness (w.r.t benchmark) in dry year (2002) (**a**), normal year (2011) (**b**), wet year (2013) (**c**) and long-term benchmark (**d**). (These maps were created using ESRI's ArcMap 10.3—https://desktop.arcgis.com/en/arcmap/, and MS-Office PowerPoint 2007 software).

The spatial distribution of RAD derived from annual deciduousness for dry, normal and wet years, respectively is given in Fig. 6a–c. The benchmark deciduousness value at each pixel was computed from 18 years of annual deciduousness (Fig. 6d) and was used to estimate RAD. In the dry year (2002), a high RAD was observed in the CDP region, and a low RAD was observed in the northern and eastern regions. In the normal year (2011), the majority of vegetation in the northern region showed a high RAD and southeastern region showed a low RAD. During the wet period (2013), a high RAD was observed all over (~ 81% of the study area) the deciduous forest dominated area than the dry (~ 65%) and normal years (~ 75%).

The area of different RAD classes in percent (ranging from 0 to 100%) is given in Table 4 and the area of RAD classes for all 18 years is given in Table S3. In the dry year (2002), the area under the highest RAD class (90–100%) was about 3.64% which increased to 5.28% in the normal year. In the wet year (2013), the highest RAD area was 7.16%. Though the RAD variations showed a clear link with rainfall pattern, the soil moisture availability also plays a crucial role. Hence, a positive relationship with rainfall in all the deciduous vegetation could not be found out.

**Table 4:** Relative annual deciduousness (in % area) during dry, normal and wet years.

RAD classes	Dry year (2002)	Normal year (2011)	Wet year (2013)
0–10	0.03	0.03	0.04
10–20	0.20	0.18	0.13
20–30	0.93	0.46	0.31
30–40	3.04	1.51	0.88
40–50	11.62	7.60	5.25
50–60	19.00	15.71	12.53
60–70	24.59	25.06	22.70
70–80	23.11	27.22	29.50
80–90	13.85	16.95	21.49
90–100	3.64	5.28	7.16

### Spatio-temporal distribution of rainfall anomalies and their frequency

**Figure 7:**
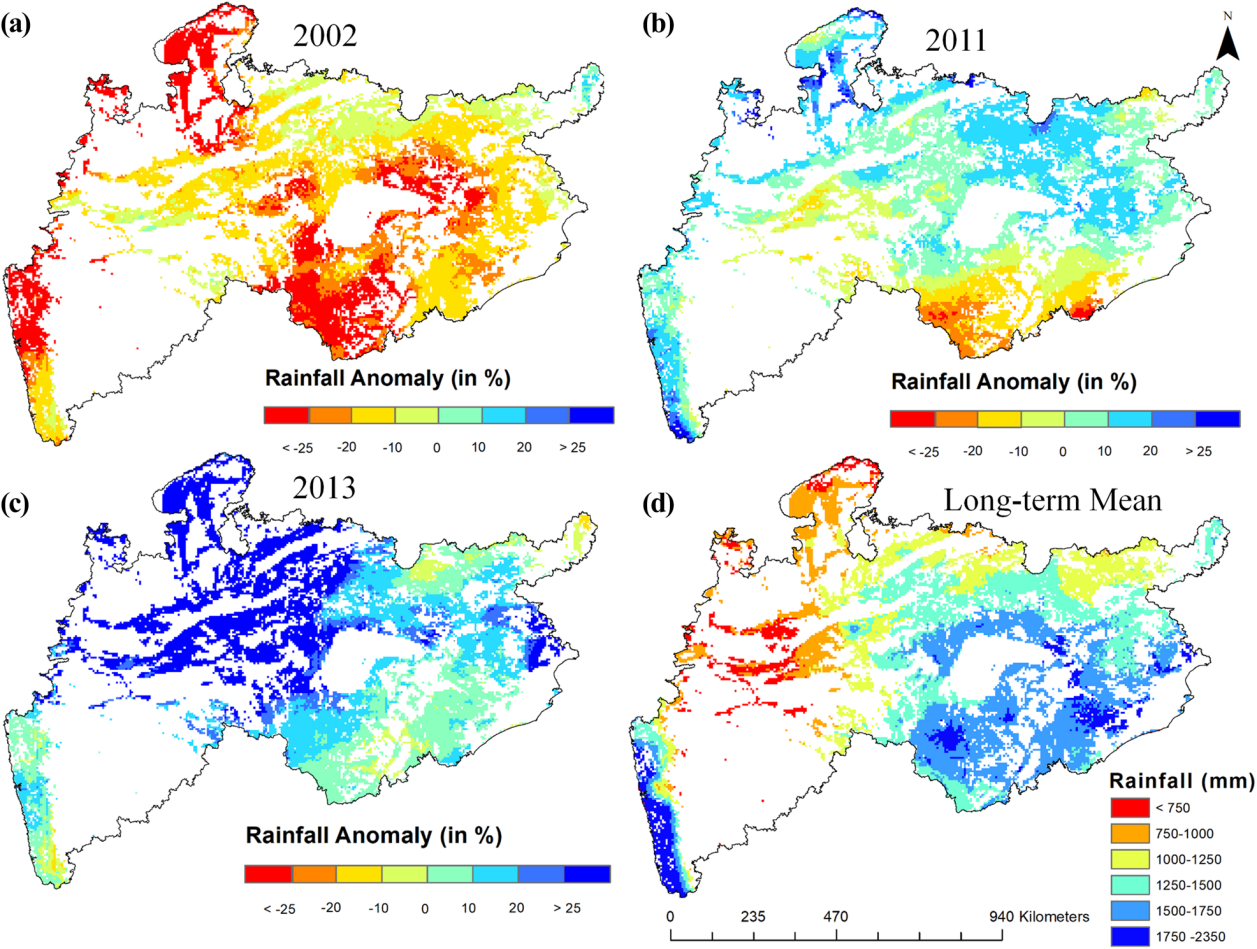
Percentage annual rainfall anomaly in dry year (2002) (**a**), normal year (2011) (**b**), wet year (2013) (**c**) and long-term mean annual rainfall (**d**). (these maps were created using ESRI's ArcMap 10.3—https://desktop.arcgis.com/en/arcmap/, and MS-Office PowerPoint 2007 software).

Figure 7a–c reveals the spatial distribution of rainfall anomalies in dry, normal and wet years, and the area statistics of these is given in Table 5. Even in the normal year some regions received low rainfall which is natural as the area is vast. The mean of the yearly rainfall anomaly of 18 years (Fig. S2) revealed that there was more negative anomaly than positive. Nearly 8% of the study area was under shortage of rainfall. Figure 7d shows the long-term average rainfall in the study area.

**Table 5:** Percent rainfall anomaly distribution (in % area) during dry, normal and wet year.

Rainfall anomaly classes	Dry year (2002)	Normal year (2011)	Wet year (2013)
< (− 25)	27.82	0.57	0.00
(− 25) to (− 20)	19.54	4.26	0.00
(− 20) to (− 10)	36.92	9.42	0.39
(− 10) to 0	14.69	15.66	4.90
0 to 10	0.82	32.68	24.96
10 to 20	0.21	32.74	24.39
20 to 25	0.00	3.34	9.06
> 25	0.00	1.33	36.29
Positive PRA	1.03	70.09	94.71
Negative PRA	98.97	29.91	5.29

To analyze the spatial distribution of extreme rainfall at different locations, we estimated the frequency of rainfall anomaly events exceeding ± 25% and ± 15% at every pixel during 2001–2018. Figure S3a,c show the spatial pattern of the frequency of positive rainfall anomaly events (> + 25% and >  + 15%), and Fig. S3b,d reveal the frequency of negative rainfall anomaly events (< − 25% and < − 15%) in the study area. Overall, the states- Madhya Pradesh (MP) and Maharashtra (MH) experienced a greater number of extreme rainfalls than other states. Around 75% of the forested area in MP and 61% of the forested area in MH experienced >  + 25% rainfall anomaly at least for twice or more. Around 35% of the forested area in MP and 33% of the forested area in MH experienced < − 25% anomaly for at least twice or more in 18 years. Overall, around 20% of the forested area in the study region was susceptible to extreme rainfall anomaly.

### Spatio-temporal distribution of deciduousness in different terrestrial ecoregions

Out of the total 15 terrestrial ecoregions (Fig. S5, classified on the basis of long-term climatic regimes and characteristic plant species^45^), only four ecoregions: (1) Narmada valley dry deciduous forests (18%), (2) Chhota-Nagpur dry deciduous forests (11%), (3) the Eastern highlands moist deciduous forests (40%) and (4) Northern dry deciduous forest (6%) have the majority of forests (~ 75%) in the study area. Therefore, these four ecoregions were only considered for examining the impact of rainfall variability on the deciduousness in different years (Figs. S4, S5).

The area percentage of different deciduousness classes for these four ecoregions are provided in the supplementary Tables S4, S5, S6 and S7. Forests under VHD class in the Eastern highlands showed a significant influence of rainfall. For the wet years 2003, 2007, 2012 and 2013, the areas estimated under VHD class were 56%, 47%, 56% and 49%, respectively, which in the dry years 2002, 2004, 2014 and 2015 reduced to 37%, 35%, 19% and 35%, respectively. The Narmada valley region experiences frequent dry period, and during 2001–2018 the region experienced 7 severe dry periods (i.e., more than 75% area was under negative rainfall anomaly). During the wet years 2005, 2011 and 2013, VHD areas were 54%, 49% and 58%, respectively, which during the dry years 2002, 2004 and 2014 reduced to 30%, 39%, and 22%, respectively in this region. However, the reduction in the deciduousness was not significant during other dry years i.e., 2009, 2017 and 2018 in this region (refer Tables S5 and S9 for details) which requires further investigation.

A random deciduousness pattern was observed in the Chhota-Nagpur plateau and the Northern dry deciduous region. In these areas, rainfall dependency was not found significant. In the Chhota-Nagpur region, the VHD class areas were 35%, 19%, 26% and 55% during the wet years 2006, 2007, 2008, and 2011, respectively and the areas were 33%, 37%, 49%, 55%, and 17% during the dry years 2002, 2005, 2009, 2010 and 2014, respectively. In this region, the year 2010 was the driest year but in the same year a high deciduousness (~ 54% of the area) was observed. In the Northern dry deciduous region, the VHD class areas were 51%, 38%, 51% and 56% during wet years 2001, 2006, 2007 and 2013, respectively and the areas were 46%, 40%, 59%, 43% and 58% during the dry years 2002, 2004,2009, 2015 and 2016, respectively. The area of VHD class remained high in the Northern dry deciduous region irrespective of rainfall variability, and it might be due to high ground water table in this region. The Chhota-Nagpur region exhibited an unpredictable deciduousness response with respect to rainfall. Rainfall anomalies in these 4 ecoregions for all the 18 years are provided in the supplementary Tables S8, S9, S10 and S11.

### Stratified analysis and sensitivity of deciduousness

Tropical forest species exhibit different phenological strategies to cope with the water shortage and leave shedding during the dry spells^46,47^. Therefore, to understand the sensitivity of deciduousness towards micro-climatic effect and physical factors, the long-term (LT) mean of the deciduousness (18 years) was grouped into four classes of the deciduousness as mentioned earlier. The area distribution of these classes were: VHD; ~ 36.85%, HD; ~ 57.84%, MD; ~ 5.12% and LD; ~ 0.19%. Vegetation type-wise variations of the long-term mean deciduousness (Fig. 4d) revealed that out of ~ 29% of the major dry deciduous forest area, around 13% of the area was under VHD and ~ 15% of the area under HD class. Out of ~ 52% of the major moist deciduous forest area, ~ 18% of the area was under VHD and ~ 32% of the area under HD class.

The forest density plays an important role in identifying the status of forest health and growth. Thus, we used Vegetation Cover Fraction (VCF) data to classify forest into three different forest density classes: (1) 0–10% as open forest, (2) 10–40% as moderately dense forest and (3) above 40% as dense forest. We analysed the deciduousness in each of the density classes, and observed that in the open density forest class, out of 34.92% of the forested area only 5.5% of the area exhibited VHD. In the case of moderate density forest class, out of 60.88% of the forested area only 31% of the area exhibited VHD. In the dense forest class, out of 4.21% of the area only 0.32% of the area exhibited VHD. The areas under HD in open, moderate and dense forest classes were 26.32%, 28.76% and 2.79%, respectively. The analysis of long-term mean deciduousness with long-term mean VCF revealed a non-linear relationship (Fig. S6, R^2^ = 0.98). In fact, the deciduousness increased with the increasing canopy density (upto VCF < 25) and then it started to decrease with the high canopy density. Also, the standard error was found to be higher in the lower density values (VCF < 40) than the higher values.

Topography plays an important role in shaping and driving the micro-habitat environment affecting species distribution, biodiversity, primary production and water resources^8,48,49^ and hence the distribution of forested area and their deciduousness in different combinations of rainfall and elevation zones was analysed. We observed that around 90.65% of the forested area is located in > 200 m above mean sea level (amsl) and 57.37% of the forested area is in > 400 m amsl elevation zones. In different elevation zones of < 300 m, 300–600 m, and > 600 m amsl, the VHD exhibited was 8.95%, 22.73% and 5.16% of the forested area, respectively. Similarly, in these three elevation zones the HD exhibited was 12.63%, 31.59% and 13.62% of the forested area, respectively. The maximum deciduousness was observed in the elevation zone of 300–600 m amsl. Around 16.01% of the deciduous forested area received low rainfall (< 1000 mm) in which 0.95% area was located in the flat (FL) regions (< 100 m), 11.01% area under medium elevated (ME) region (200–500 m), 3.7% area under high elevated (HE) region (500–800 m) and 0.88% area under very high elevated (VHE) region (> 800 m). Around 42.95% of the forested area received moderate rainfall (1000–1500 mm) with the area distribution of 2.45%, 24.74%, 10.62% and 5.14% in different elevation zones like FL, ME, HE and VHE, respectively (Table S12). Around 41.04% of the area was under high rainfall region (> 1500 mm) with the area distribution of 5.44%, 19.65%, 10.51%, and 5.43% under FL, ME, HE and VHE zones, respectively. Out of all the forested area around 95% of the area exhibited high to very high deciduousness in which 35.24% area was observed in the elevation zone of > 500 m amsl.

## Discussion

Partitioning the ecosystem in terms of their goods and services especially for carbon sequestrations and water fluxes is an important issue across the world^13,50–52^. Though, the timing of start of greening and end of senescence may not vary drastically in a short-term period in the tropical forests^53^, deciduousness is sensitive to intra and inter-annual micro-climatic variations. Hence, we considered deciduousness as a quasi-indicator to study the impact of extreme rainfall conditions on leaf-fall variability. The majority of deciduous vegetation (~ 54%) was observed only in the Central Indian states- Madhya Pradesh and Chhattisgarh, followed by 36% in Maharashtra (southern Indian state) and Odisha (eastern Indian state). The very high deciduousness was observed dominantly along the border regions of Chhattisgarh, Maharashtra and Madhya Pradesh states (Figs. 4 and 6). Most of the previous studies carried out on the deciduous forests in the study region are either on litter fall variability^54,55^ or phenology^56–58^. However, these studies were carried out through field observations in smaller areas and for shorter temporal periods. Few studies have tried to understand the vegetation response using remote sensing satellite data^38,53,59,60^. In contrast, this study revealed comprehensible yearly differences in magnitude and intensity of deciduousness and its significant link with the rainfall variability at different locations of the study area.

According to Elliot et al.^61^, Williams et al.^62^, and Felton and Smith^63^, the inter-annual variation in the deciduousness was due to physiology, soil–water availability, rainfall, temperature, and relative humidity. In this sense, the spatio-temporal variation in the deciduousness as revealed in this study will help the ecologists to detect the presence of different plants species, relate with species-specific physiology, soil moisture regimes, and landscape resilience. It will also assist in prioritizing the sampling areas for ground-based survey.

Kunhamu et al.^64^ observed that the deciduousness varied linearly with the density of trees. They also found the reduction of leaf-fall from 11.18 to 5.73 Mg ha^−1^ with decrease of the forest density to a two-third level. However, the present study observed a linear relationship of the deciduousness with the forest density only in the low forest density classes (Fig. S6), not in the high density classes. Also, the study conducted by Jeganathan et al.^65^ revealed considerable differences in the VCF values with FSI published forest density maps in two different forest locations in India. Moreover, the VCF values were not found as the actual representation of the forest density classes. The VCF underestimated both the low and high forest density classes considerably (Fig. S7). Thus, it is suggested that the non-linear relationship between deciduousness and VCF may further be scrutinized and carefully interpreted for Indian forests. The explanation for this observation could be the inclusion of mixed pixels of non-deciduous vegetation types (i.e., presence of evergreen or semi-evergreen vegetation type within the pixel) in the deciduous forest map. According to Singh and Kushwaha^66^ the *Shorea robusta* (a dominant species in India) has a paradoxical nature and suggested to consider this species under semi-evergreen type. However, the vegetation type map used in this study considered S*horea robusta* under deciduous forest (Roy et al.^67^ 2015), which might have resulted the low deciduousness in high VCF causing a non-linear relationship.

In terms of vegetation growth, two contradicting observations exist in the literature. Condit et al.^68^ and Duan et al.^69^ observed that the magnitude of growth was affected by water availability. While, Elliot et al.^61^ observed that the leaf flushing relied on sub-surface water availability and the rainfall periodicity alone is not the principle determinant in all places of the tropical forests in many parts of the Asia. Interestingly, in this study, we observed the effect of both these factors on the vegetation growth through the deciduousness (Figs. 3 and 4). For example, the area under VHD was 34.38% in the drought year (2002), which increased to 49.67% in the next year (2003), a high rainfall year. Similarly, there was a clear increase of the deciduousness (51.06%) in the wet year (2013) than (42.38%) in the normal year (2011). The year 2006 received high rainfall in the Central India but the areal extent of VHD class was only 25%. It reflected the state of dryness, and this was due to the low rainfall in the forest dominant areas. On the other hand, the positive productivity and high deciduousness observed in the drought years (such as 2009) could only be explained properly through the soil moisture information, which can be taken up in our future study. The deciduous vegetation occurring in the lower catchment portion was observed to be independent of rainfall, as the rainfall in the upper catchment invariably brings water downwards. Hence, one should be careful in interpreting rainfall dependencies in such cases.

Further, a high rainfall does not guarantee a high moisture in a region as soil moisture depends on variety of factors such as altitude, slope, soil and its water holding capacity, drainage density, surface runoff, evapotranspiration, and vegetation types. On the other hand, even in the case of shortage of rainfall, presence of high soil moisture due to run off from upper catchment might support the vegetation growth. Overall, high temperature with low rainfall in a poor water-holding soil impacts the magnitude of growth in the study region^70^. It is interesting to note that though ~ 84% of this region received more than 1000 mm of annual rainfall, this landscape is dominated by deciduous vegetation. It is because the mean annual potential evapotranspiration in this region is very high (> 1250 mm)^71^, and hence susceptible to frequent dry periods. Several studies have reported a time-lag between vegetation response to precipitation^72–74^. It is interesting to note that nearly 50% of the peak growth of deciduous vegetation in the Central India is attained in the beginning of the monsoon period, and during the rainfall period vegetation performs accelerated photosynthesis and biomass accumulation using the abundant sunlight availability in this region. In case of deficiency in rainfall, it gets back to its adaptation mechanism and stops its activities thereby reducing the amplitude of growth. This results in reduction of the deciduousness index.

## Conclusion

The detailed multi-level testing was carried out in this study to reveal the performance of old and new metric and sensitivity of the deciduousness with rainfall variability over 18 years. Around 95% of the forested area in the Central India is of deciduous type, out of which around 36% of the forested area exhibited the very high deciduousness. Most of the study area consisting of the deciduous vegetation showed a positive relationship of the deciduousness with rainfall. However, the regions having evergreen, semi-evergreen and good ground water availability showed spatial variability in this relationship. The VHD class of deciduousness observed was low (34.38%) in the dry year (2002), moderate (42.38%) in the normal year (2011) and high (51.06%) in the wet year (2013).

Under the low (< 1000 mm) rainfall region, the deciduousness was lower (VHD area: 4.75% and HD area: 9.56%) than the high (> 1500 mm) rainfall region (VHD area: 16.14% and HD area: 22.89%). The Narmada valley region experienced 7 dry periods during 2001–2008. Forests in the Narmada Valley and Eastern highlands regions revealed a significant rainfall dependency. The Chhota-Nagpur plateau and Northern dry deciduous regions revealed a random deciduous pattern, and rainfall dependency was not found significant.

A positive relationship between forest density and the deciduousness was observed only in the low density classes. Further, the VCF values were not the actual representation of the forest density classes. In fact, the VCF underestimated both the low and high forest density classes considerably. In terms of topographic elevation, the HD and VHD classes (54% of the forested area) were observed mainly in the elevation zone of 300–600 m amsl.

Thus, it can be stated that the present study improved the spatial–temporal understanding of the deciduousness and its sensitivity to climatic extremes. This will help the ecologists to carry out further detailed ground survey in the sensitive zones. The findings of this study contribute towards the objectives of Long-Term Ecological Observatories (LETO) Programme initiatives launched in 2015 by the Indian Government to study and monitor health of the Central Indian forests. The information about the deciduousness behaviour at landscape will help forest managers to demarcate fire-prone zones. Forest fire models around the world can include the deciduousness as one of the parameters for predicting spatio-temporal spread of forest fire. Since, the deciduousness is the indicator of leave shedding phenomena, our study will help to model litter fall across the tropical deciduous forest. This in turn would help in understanding the vegetation dynamics and nutrients recycling of this ecosystem.

## Material and methods

### Study area

The study region i.e. the Central Indian forested landscape (Fig. 1) covers 5 Indian states—Maharashtra, Madhya Pradesh, Chhattisgarh, Odisha and Jharkhand. As per Forest Survey of India's (FSI) report^75^, the Indian subcontinent contains around 21.54% (708,273 km^2^) of forest cover (excluding trees outside the forested areas), and the Central Indian forest cover is about 7.86% (258,541 km^2^). The total geographical area of these five states is 986,580 km^2^ and 26.20% of area consists of forest cover^75^. This tropical forested landscape exists amidst extensive human pressure. This landscape provides shelter for diverse life forms through 108 wildlife sanctuaries, 22 national parks 17 tiger reserve and 8 elephant reserves. Also, this landscape houses a series of protected forests connecting corridors for tigers and elephants which are meant to maintain the gene flow, species, and wildlife movement^32^.

The Central India receives its majority of rainfall (~ 80%) during southwest monsoon (June–September), and temperature varies from a minimum of 15 °C in winter to a maximum of 45 °C in summer. This landscape is under huge pressure from anthropogenic activities such as use of forest for fuel, fodder, timber, medicine, and other ecological services. The Central Indian forest landscape provides vast opportunities for researchers to take up diverse studies. As per FSI (2017), India's carbon stock was estimated to be 7083 million tons (Mt), and stock among major forest types like dry deciduous, moist deciduous, evergreen and semi-evergreen forest was 2670.34, 1323.02, 328.07 and 1124.43 Mt, respectively^75^. The distribution of carbon stock in different states such as Maharashtra, Madhya Pradesh, Chhattisgarh, Odisha and Jharkhand of the study area was 493.02, 695.66, 560.98, 452.90, and 222.88 Mt, respectively.

### MODIS data

Moderate Resolution Imaging Spectroradiometer (MODIS)—MOD13Q1 (Collection 6) Terra composite NDVI data (at 16 days interval and 250 m spatial resolution) were downloaded over 18 years (2001–2018) from LPDAAC (Land Processing Distributed Active Archive Center (https://lpdaac.usgs.gov). The MODIS standard VI products are atmospherically corrected for bi-directional surface reflectance and are masked for water, clouds, heavy aerosols, and cloud shadows^76,77^.

### Climate hazards group infrared precipitation with station (CHIRPS) data

The CHIRPS data is the longest archive of precipitation datasets available at a global level which spans for more than 30 + years on a monthly basis^78^. The data spans from 50° S–50° N latitudes and are available for all longitudes from 1981 onwards. For this study CHIRPS monthly datasets were downloaded for a period of 18 years (ftp://ftp.chg.ucsb.edu/pub/org/chg/products/CHIRP/monthly/). The CHIRPS data version 2.0 at a spatial resolution of 0.05° (~ 5 km) was used in this study. The CHIRPS is the product of a collaborative project taken by the scientists working in US Geological Survey (USGS) and Earth Resources Observation and Science (EROS) for early warning system, and were created using precipitation estimates from satellite imagery merged with in-situ gauging stations data as a gridded rainfall time series^79^.

### Other datasets

MODIS Vegetation Continuous Field (VCF) (MOD44B v006) and ASTER Global Digital Elevation Model (ASTGTM v003) products were also used in this study (see https://lpdaac.usgs.gov for more details). The annual VCF product provides a percent tree cover information i.e., the total canopy of trees having 5 m or greater height, at a spatial resolution of 250 m across the globe (see https://lpdaac.usgs.gov/products/mod44bv006/ for more details). The VCF data helped to analyse the distribution of the deciduousness under different canopy cover categories. The DEM from ASTER is available at one arc second spatial resolution over 83° North to 83° South. This data helped to quantify the presence of forest and the deciduousness at different elevation zones. The most comprehensive vegetation type map prepared for the Indian subcontinents so far was by Roy et al.^67^ at 1: 50,000 scale using the LISS III sensor (23.5 m) (https://bis.iirs.gov.in). From this map, we extracted all the classes of dry deciduous, moist deciduous, semi-evergreen, and evergreen forest types and converted all these classes into one single forest class. Then, this forest map was up-scaled to 250 m using threshold based aggregation procedure. For this purpose, the percentage of forest pixels (of size 23.5 m) occurring within each of the 250 m MODIS grids was estimated, and the grids having ≥ 75% forest cover only were marked as forested grid. The final forest map derived at 250 m grid was used as forest mask for further analysis.

Terrestrial Ecoregions of the World (TEOW) is a biogeographic regionalization of the Earth's terrestrial biodiversity defined as relatively large units of land or water containing a distinct assemblage of natural communities sharing a large majority of species, dynamics, and environmental conditions^45^. The terrestrial ecoregion map of the world was downloaded from Data Basin website (https://databasin.org/datasets/). Out of the total 51 terrestrial ecoregions of India, 15 ecoregions falling within the study area were extracted and utilised in this study (Fig. S5).

### Time-series data processing

The study area is covered by 6 MODIS tiles (i.e. h24v06, h24v07, h25v06, h25v07, h26v06, h26v07), and a total of 2484 images were acquired for 18 years (2001–2018). The overall methodology followed in this research is depicted in Fig. S8. The pixels having reliability flags 0 and 1 were only used for further analysis, while the rest of the unreliable pixels were discarded. The composites of all the tiles for each year were mosaicked, and only the forested pixels were processed.

At first, we filled the gaps, created due to the elimination of unreliable pixels, using appropriate noise-free temporal neighborhood values to keep the phenological trend unchanged. Then, we eliminated the erroneous values at each pixel using the statistical outlier removal technique^55,56,79^. The noise eliminated data sets were then smoothed using Discrete Fourier Transform (DFT) with initial six harmonics^38,80^.

### Estimation of vegetation growth

#### Quantifying deciduousness

According to White and Nemani^81^ and Cuba et al.^14^, a pixel is dominantly deciduous if the amplitude (i.e. difference between minimum and maximum) of yearly VI profile in a pixel is greater than 50% of annual maximum (Fig. 2). We observed that this approach is falsely detecting the smaller height vegetation types (i.e., pheno-classes 3 and 4 in Fig. 2c) as highly deciduous. The following two equations (Eqs. 1, 2) were used to estimate the annual deciduousness (AD) which reveals the remote sensing derived proxy leaf fall percentage in a pixel. The Eq. (1) was given by Cuba et al.^14^ and the improved new metric is given in the Eq. (2).

1 $$Annual \,\,Deciduousness\,\, \left( {Old} \right)_{i,x,y} = \frac{{Max_{i,x,y} - Min_{i,x,y} }}{{Max_{i} ,x,y}} \times 100$$

2 $$Improved \,\,Annual\,\, Deciduousness\,\, \left( {AD} \right)_{i,x,y} = \frac{{Max_{i,x,y} - Min_{i,x,y} }}{{Max_{i} ,x,y}} \times \frac{{Mean_{i,x,y} }}{{S_{c} }}$$

where, *i* is the year, and (x, y) are the column and row positions. Min_*i,x,y,*_, Max_*i,x,y*_ and Mean_*i,x,y*_ are the minimum, maximum and mean NDVI value of a pixel at (x, y) in *i*th year, respectively. S_c_ is the scaling constant (S_c_ = 0.005). Expected values of AD i.e. theoretical minimum and maximum will be in the range of 0–100.

The parameters involved in the deciduousness estimation are depicted graphically using an illustrative theoretical annual profile (Fig. 2a). A comparison of both the metrics was made using actual RS derived NDVI values from moist and dry deciduous samples (Fig. 2b) and 4 theoretical pheno-classes (Fig. 2c). The performance of the new and old metric was checked in different vegetation classes having high to low deciduousness categories using well distributed samples across the study area. If we consider deciduousness as a proxy litter fall, RS based deciduousness estimates must be relatively similar to the ground-based litter fall observation. In this regard, litter fall from different vegetation types in the study region was collected through literature review and was used to check the results from both the metrices. The degree of magnitude of growth in terms of deciduousness of tropical forest was examined during 2001–2018. In this study, unlike Cuba et al.^14^, we mapped and examined the variability of deciduousness in different climatic extremes and strata without applying any threshold value.

In order to compare the deciduousness estimated for different years within the study area, and to understand the climatic impact, we estimated relative annual deciduousness (RAD). But for this, we needed a reference benchmark deciduousness value. Thus, we considered the benchmark AD as the highest AD value at a pixel out of 18 years (referred as MaxAD_*LT*_; LT is Long-Term). For the study area we needed a single representative benchmark deciduousness and hence we considered mean of MaxAD_*LT*_ of the entire study area. Finally, RAD was calculated by using the Eq. (3) where the constant 100 is used to convert deciduousness into a percentage.

3 $$Relative \,\,Annual\,\, Deciduouness\left( {RAD} \right)_{i,x,y} = { }\frac{{AD_{i,x,y} }}{{Mean\left( {MaxAD_{LT} } \right)}} \times { }100$$

The denominator is considered as the benchmark deciduousness for the study area. RAD is useful in understanding the AD variability in dry, wet and normal years.

### Rainfall anomaly

There is no dearth of sunlight availability in the Central India, so the variation in the deciduousness would mainly depend upon the water availability. Therefore, to understand the variation in water availability in different years, spatial–temporal variability in the rainfall anomaly was estimated based on annual accumulated rainfall (AAR) for the years 2001–2018.

4 $${\text{Percentage }}\,\,{\text{Rainfall }}\,\,{\text{Anomaly }}\left( {{\text{PRA}}} \right)_{i} = \frac{{AAR_{i} - AAR_{LT} }}{{AAR_{LT} }} \times 100$$

where, AAR_*i*_ represents Annual Accumulated Rainfall in *i*th year and AAR_*LT*_ represents the long-term mean AAR over 18 years. PRA was estimated at every pixel and used to check whether a year is a dry, wet or normal year. Also the correlation between AD and rainfall during 2001–2018 was estimated at every pixel.

### Stratified spatial analysis of deciduousness

The deciduousness of the landscape was analyzed for its variability with rainfall at different stratification levels: (a) Vegetation types, (b) Altitudinal zones (c) Vegetation Continuous Fraction and (d) Ecoregions wise.

### Analysis of variance

In order to test the intra and inter year variability in the deciduousness values derived from (a) the old and new metric, (b) in dry and moist vegetation type and (c) in dry, wet and normal years, we utilized ANOVA statistics. Around 800 random samples were taken from the deciduous vegetated class (dry and moist forest types) and divided into 2 sample groups: first group was taken over pixels in the open to moderately dense forest having VCF density range of < 30% and the second group was taken over pixels in the dense forest having VCF > 40%.

## Supplementary information

Supplementary file 1

## Data Availability

All the spatial datasets used in this are available in the web for downloading free of charge. All the resultant data used in this study are available from the authors upon request.
