# Supplementary material for: Improved NDVI based proxy leaf-fall indicator to assess rainfall sensitivity of deciduousness in the central Indian forests through remote sensing
Source: Sci Rep. 2020 Oct 19;10:17638. doi: 10.1038/s41598-020-74563-2 (PMC7572383; doi:10.1038/s41598-020-74563-2)
Supplement: Supplementary file 1 [file 41598_2020_74563_MOESM1_ESM.docx]

**Improved NDVI based proxy leaf-fall indicator to assess rainfall sensitivity of deciduousness in the central Indian forests through remote sensing**

**Beependra Singh, C. Jeganathan* and V. S. Rathore**

Department of Remote Sensing

Birla Institute of Technology (BIT), Mesra

Ranchi, Jharkhand, India

* Corresponding Author

**Autors Affiliations:**

1. **Mr. Beependra Singh**,

Researcher

Department of Remote Sensing, Birla Institute of Technology (BIT), Mesra, Ranchi 835215, Jharkhand, India.

Tel.: +91 9632929011; e-mail: beependrasingh@yahoo.com

2. **Dr. C. Jeganathan***,

Professor & Head

Department of Remote Sensing, BIT, Mesra, Ranchi, Jharkhand, India

Tel.: +91 8987630041; e-mail: jeganathanc@bitmesra.ac.in

3**. Dr. V. S. Rathore,**

Assistant Professor,

Department of Remote Sensing, BIT, Mesra, Rnachi, Jharkhand, India.

Tel.: +91 9431382641; e-mail: vsrathore@bitmesra.ac.in

**List of Supplementary Figures**

**Figure S1.** NDVI annual profile of 22 sample points from the normal year (2011) in different vegetation types: (a) Moist deciduous, (b) Dry Deciduous, (c) Semi-Evergreen and (d) Evergreen. (Note: The spatial locations of these sample points are marked in Fig. 1).

**Figure S2.** Inter-annual variability of Percentage Rainfall Anomaly (2001 - 2018).

**Figure S3.** Spatial distribution of frequency of rainfall anomaly observed during 2001 to 2018: (a) positive anomaly (>+25%), (b) negative anomaly (< -25%), (c) positive anomaly (>+15%), (d) negative anomaly (< -15%).

**Figure S4.** Percentage rainfall anomaly in different terrestrial ecoregions during 2001 to 2018.

**Figure S5.** Spatial extent of terrestrial ecoregions in the study area.

**Figure S6.** Variation of long-term (18 years) mean deciduousness with long-term mean VCF.

**Figure S7.** Differences in the VCF values with ground forest density.

**Figure S8.** Methodology.

**List of Supplementary Tables**

Table S1. Better performance of the new metric for different pheno-classes.

Table S2. Inter-annual area distribution of different categories of Deciduousness in the Central Indian landscape (2001-2018).

Table S3. Inter-annual area distribution of different categories of Relative Deciduousness (2001-2018).

Table S4. Deciduousness variations in Chhota-Nagpur ecoregion in different years (2001 to 2018).

Table S5. Deciduousness variations in Narmada Valley ecoregion in different years (2001 to 2018).

Table S6. Deciduousness variations in Eastern Highlands ecoregion in different years (2001 to 2018).

Table S7. Deciduousness variations in Northern Dry Deciduousness ecoregion in different years (2001 to 2018).

Table S8. Percentage Rainfall Anomaly (PRA) in Chhota-Nagpur ecoregion.

Table S9. Percentage Rainfall Anomaly (PRA) in Narmada Valley ecoregion.

Table S10. Percentage Rainfall Anomaly (PRA) in Eastern Highlands ecoregion.

Table S11. Percentage Rainfall Anomaly (PRA) in Northern Dry Deciduous ecoregion.

Table S12. Deciduousness distribution (in % area) under different elevation zones in the study area.

[Note: All the values are in % area.]

**
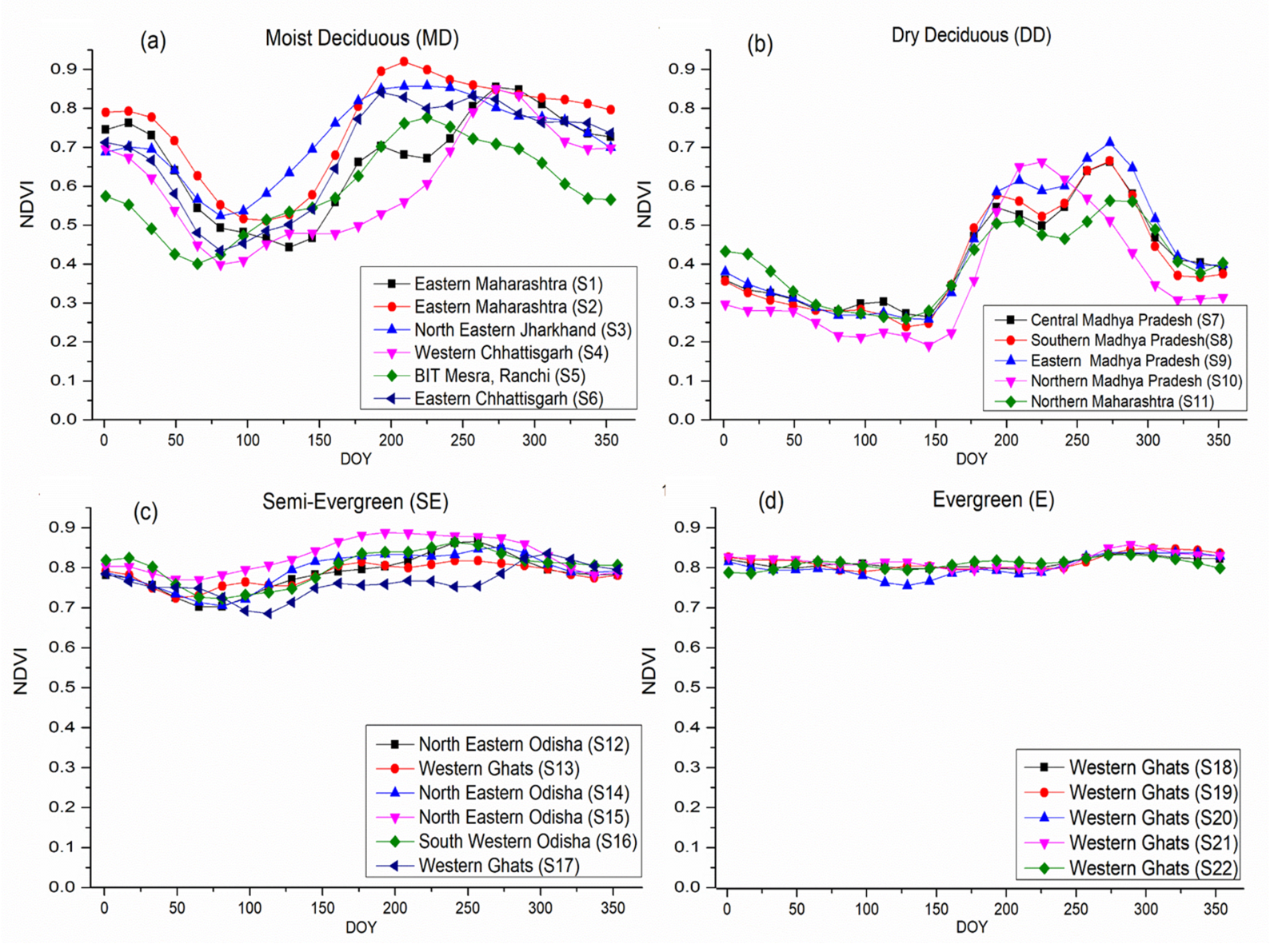
**

**Figure S1.** NDVI annual profile of 22 sample points from the normal year (2011) in different vegetation types: (a) Moist deciduous, (b) Dry Deciduous, (c) Semi-Evergreen and (d) Evergreen. (Note: The spatial locations of these sample points are marked in Fig. 1).


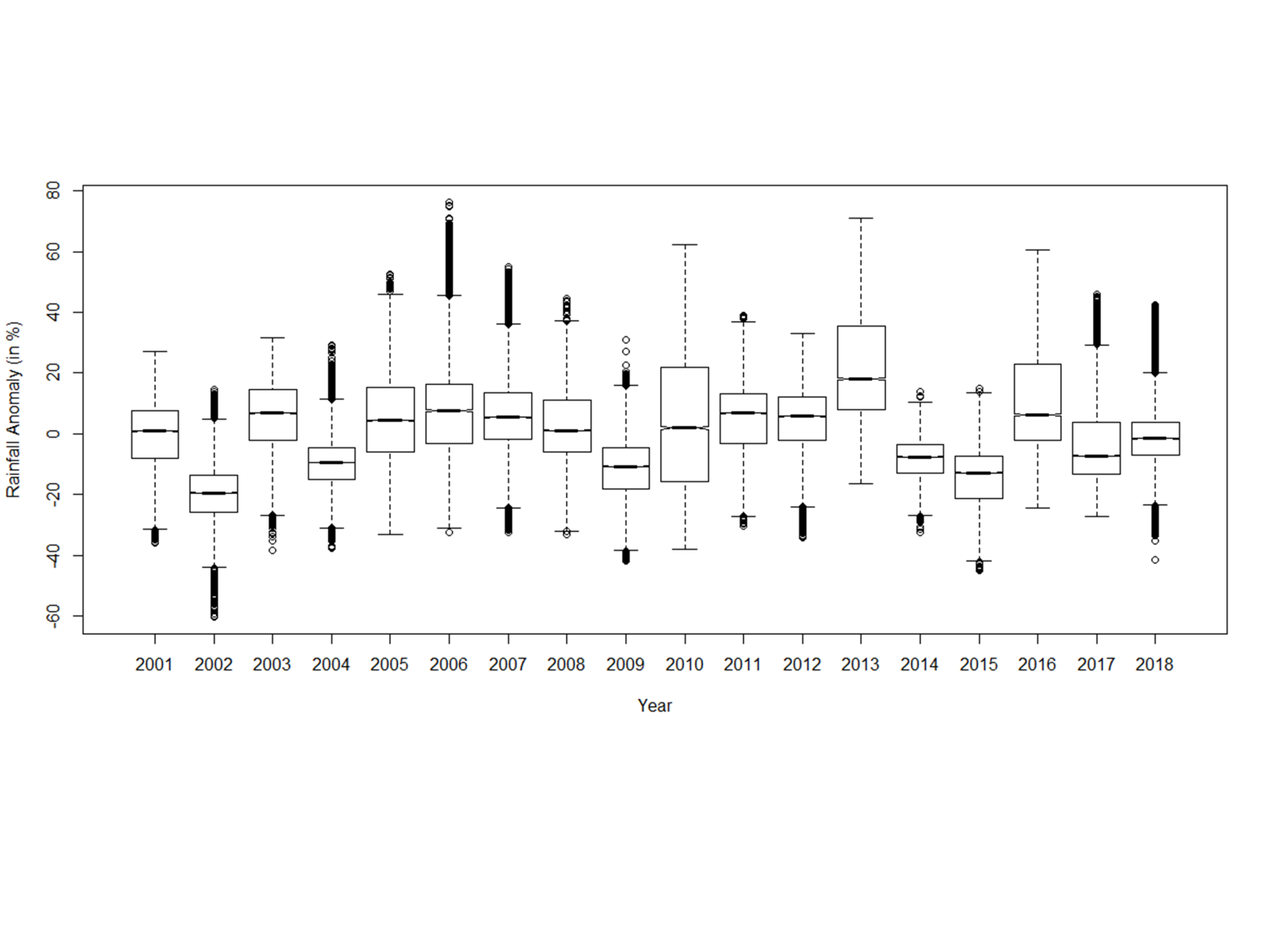


**Figure S2**. Inter-annual variability of percentage rainfall anomaly (2001 - 2018).


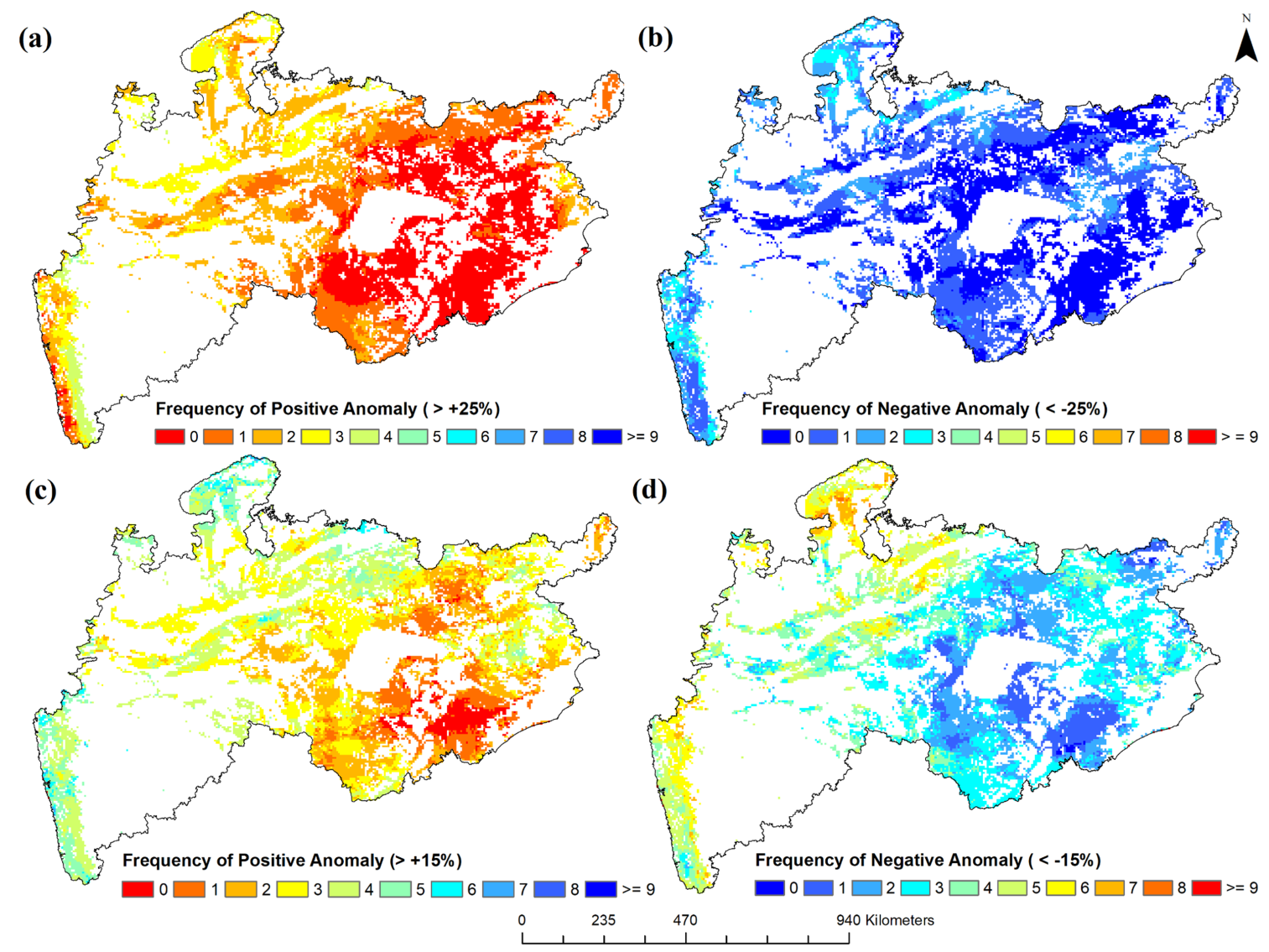


**Figure S3**. Spatial distribution of frequency of rainfall anomaly observed during 2001 to 2018: (a) positive anomaly (>+25%), (b) negative anomaly (< -25%), (c) positive anomaly (>+15%), (d) negative anomaly (< -15%). (These maps were created using ESRI's ArcMap 10.3 - https://desktop.arcgis.com/en/arcmap/, and MS-Office PowerPoint 2007 software).


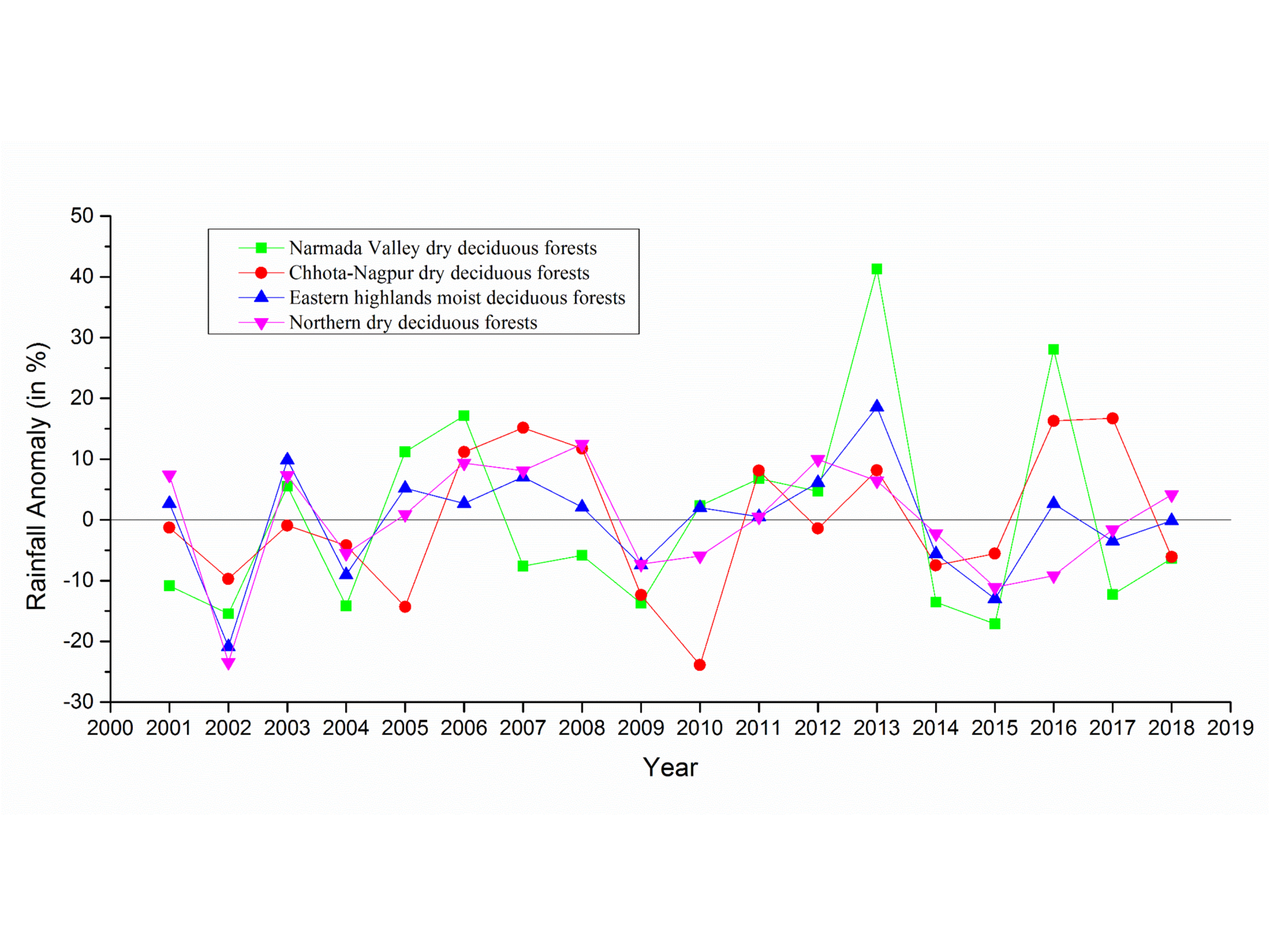


**Figure S4.** Percentage rainfall anomaly in different terrestrial ecoregions during 2001 to 2018.


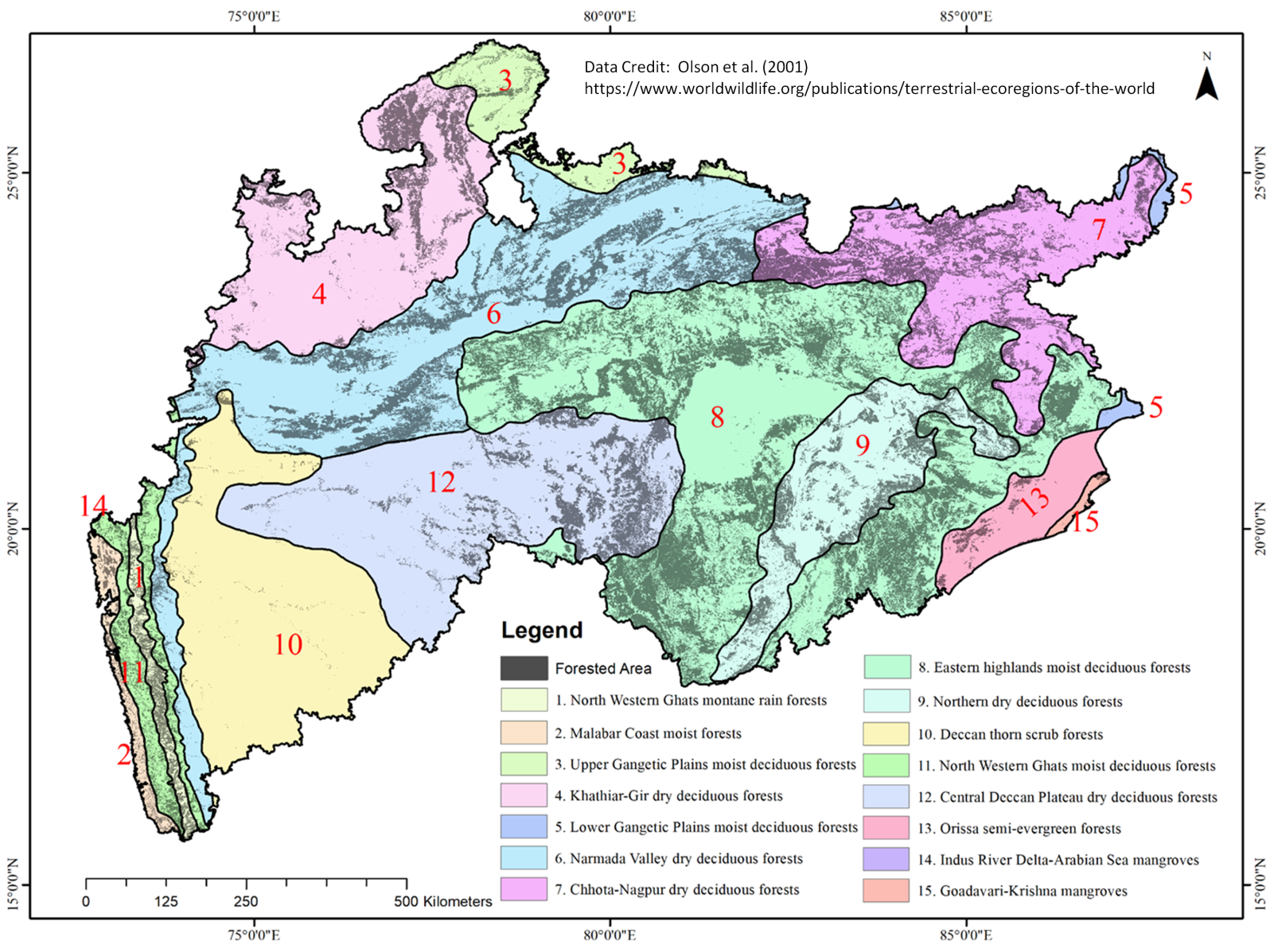


**Figure S5**. Spatial extent of terrestrial ecoregions in the study area. (This map was created using ESRI's ArcMap 10.3 - https://desktop.arcgis.com/en/arcmap/, and MS-Office PowerPoint 2007 software).


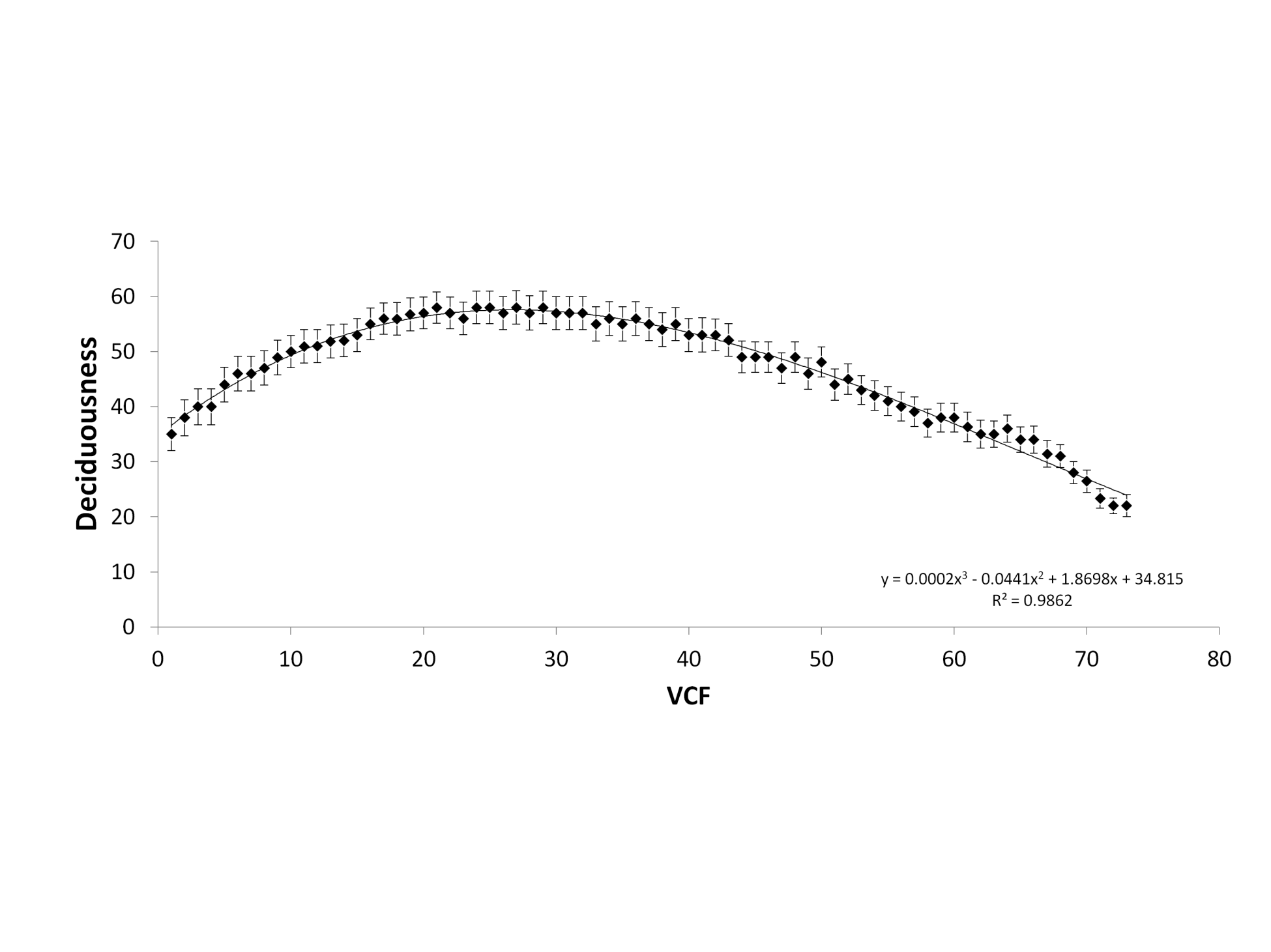


**Figure S6.** Variation of long-term (18 years) mean deciduousness with long-term mean VCF.


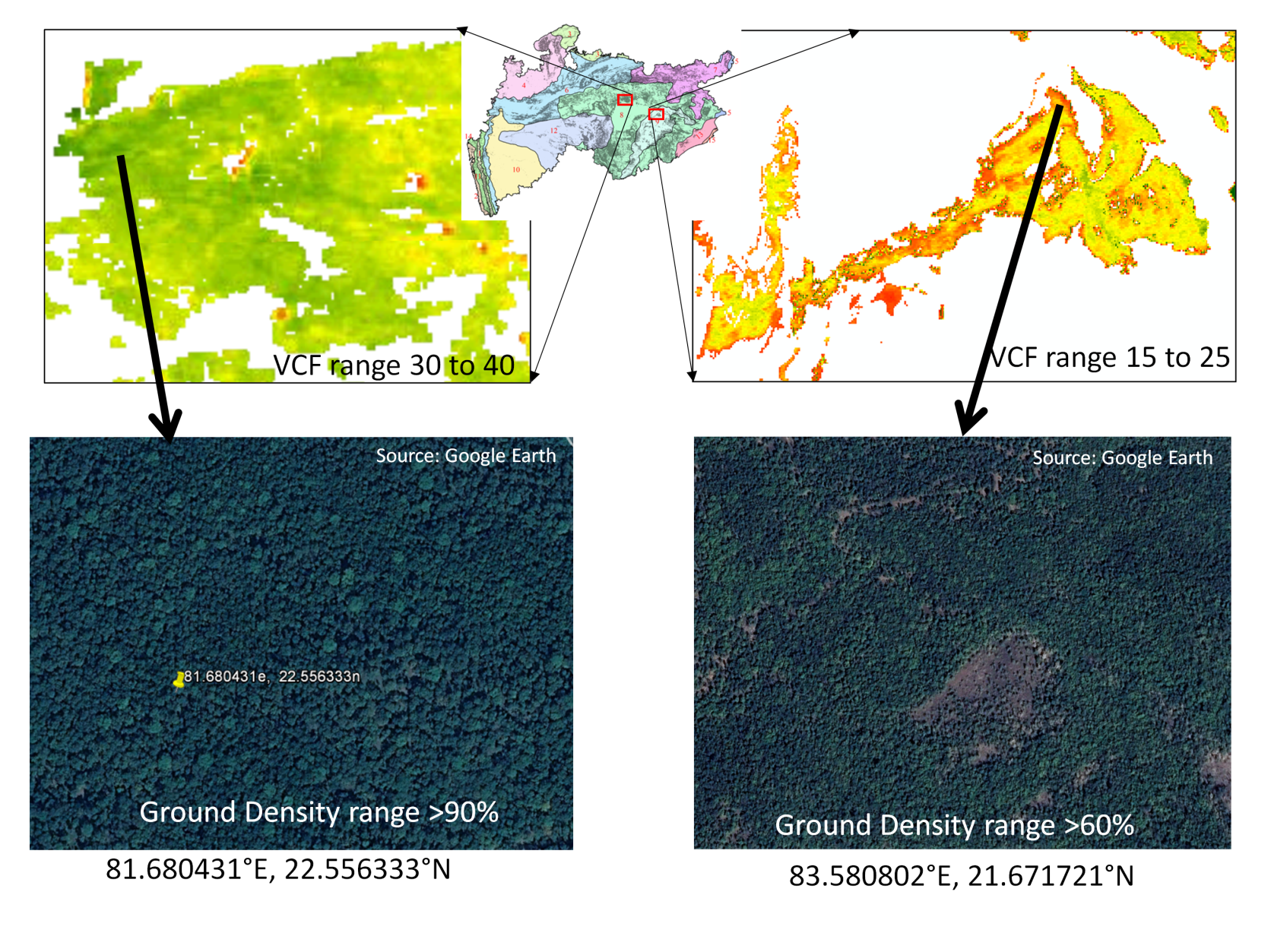


**Figure S7.** Differences in the VCF values with ground forest density. (These figures were created using ESRI's ArcMap 10.3 - <https://desktop.arcgis.com/en/arcmap/>, Google Earth - <https://www.google.com/intl/en_in/earth/>, and MS-Office PowerPoint 2007 software).


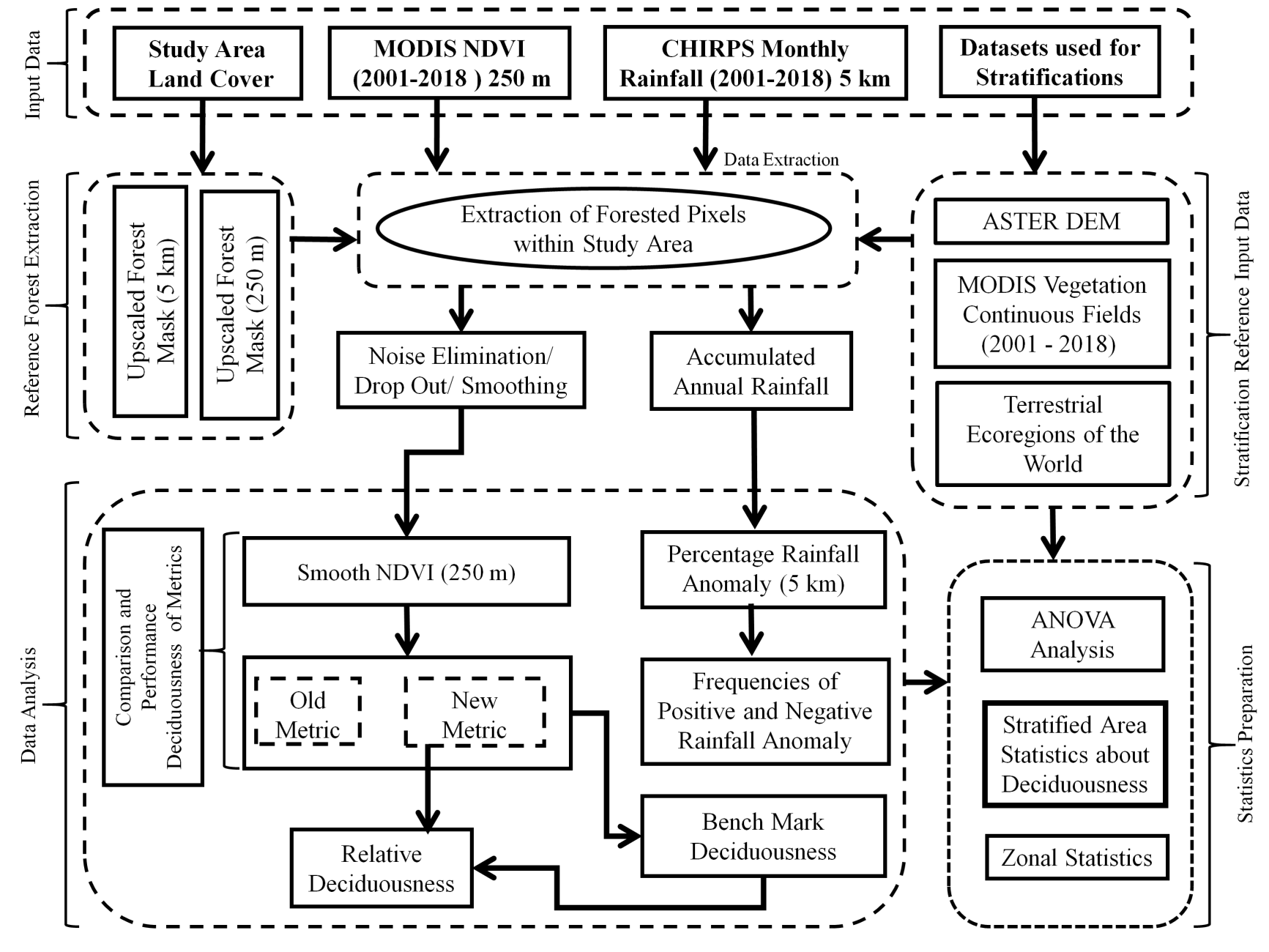


**Figure S8**. Overall Methodology.

**Table S1.** Better performance of the new metric for different pheno-classes.

| **Vegetation Group** | **MIN** | **MAX** | **MEAN** | **Old Metric** | **New Metric** |
| --- | --- | --- | --- | --- | --- |
| Pheno-class 1 | 0.35 | 0.85 | 0.6 | = [(0.85-0.35)/0.85 * 100  = **58.82** | =[ (0.85-0.35)/(0.85)] * (0.6/0.005)  = **70.59** |
| Pheno-class 2 | 0.25 | 0.65 | 0.45 | = [(0.65-0.25)/0.65] * 100  = **61.54** | = [ (0.65-0.25)/0.65] * (0.45/0.005)  = **55.38** |
| Pheno-class 3 | 0.2 | 0.5 | 0.35 | = [(0.5-0.2)/0.5] * 100  = **60** | = [ (0.5-0.2)/0.5] * (0.35/0.005)  = **42** |
| Pheno-class 4 | 0.1 | 0.25 | 0.17 | = [(0.25-0.1)/0.25] * 100  = **60** | =[(0.25-0.1)/0.25] * (0.17/0.005)  = **20.4** |

**Table S2.** Inter-annual area distribution (in %) of different categories of Deciduousness in the Central Indian landscape.

| Class / Year | 2001 | 2002 | 2003 | 2004 | 2005 | 2006 | 2007 | 2008 | 2009 | 2010 | 2011 | 2012 | 2013 | 2014 | 2015 | 2016 | 2017 | 2018 |
| --- | --- | --- | --- | --- | --- | --- | --- | --- | --- | --- | --- | --- | --- | --- | --- | --- | --- | --- |
| LD | 0.36 | 0.54 | 0.31 | 0.23 | 0.25 | 0.27 | 0.54 | 0.43 | 0.20 | 0.27 | 0.38 | 0.23 | 0.30 | 0.41 | 0.38 | 0.40 | 0.30 | 0.39 |
| MD | 8.18 | 11.94 | 4.93 | 8.13 | 6.01 | 10.73 | 7.91 | 9.21 | 3.79 | 4.09 | 7.00 | 4.73 | 4.57 | 11.82 | 7.66 | 6.42 | 4.40 | 6.51 |
| HD | 52.67 | 53.14 | 45.08 | 56.33 | 50.36 | 63.11 | 50.69 | 58.03 | 42.37 | 37.75 | 50.24 | 46.11 | 44.08 | 66.62 | 56.79 | 47.96 | 40.38 | 48.14 |
| VHD | 38.8 | 34.38 | 49.67 | 35.31 | 43.37 | 25.9 | 40.85 | 32.32 | 53.63 | 57.9 | 42.38 | 48.92 | 51.06 | 21.14 | 35.16 | 45.21 | 54.91 | 44.95 |

**Table S3.** Inter-annual area distribution (in %) of different categories of Relative Deciduousness.

| Class / Year | 2001 | 2002 | 2003 | 2004 | 2005 | 2006 | 2007 | 2008 | 2009 | 2010 | 2011 | 2012 | 2013 | 2014 | 2015 | 2016 | 2017 | 2018 |
| --- | --- | --- | --- | --- | --- | --- | --- | --- | --- | --- | --- | --- | --- | --- | --- | --- | --- | --- |
| 0-10 | 0.04 | 0.03 | 0.03 | 0.02 | 0.02 | 0.02 | 0.04 | 0.04 | 0.02 | 0.02 | 0.03 | 0.03 | 0.04 | 0.06 | 0.04 | 0.05 | 0.03 | 0.03 |
| 10-20 | 0.14 | 0.20 | 0.13 | 0.09 | 0.12 | 0.11 | 0.23 | 0.19 | 0.09 | 0.13 | 0.18 | 0.11 | 0.13 | 0.19 | 0.18 | 0.19 | 0.13 | 0.18 |
| 20-30 | 0.54 | 0.93 | 0.41 | 0.43 | 0.33 | 0.46 | 0.71 | 0.58 | 0.24 | 0.30 | 0.46 | 0.24 | 0.31 | 0.51 | 0.48 | 0.44 | 0.39 | 0.46 |
| 30-40 | 1.97 | 3.04 | 1.14 | 1.83 | 1.24 | 2.25 | 1.80 | 2.09 | 0.82 | 0.94 | 1.51 | 0.89 | 0.88 | 2.47 | 1.70 | 1.43 | 1.15 | 1.38 |
| 40-50 | 8.45 | 11.62 | 5.23 | 8.80 | 6.83 | 11.70 | 8.28 | 9.73 | 4.15 | 4.32 | 7.60 | 5.51 | 5.25 | 13.01 | 8.29 | 6.87 | 4.33 | 7.16 |
| 50-60 | 16.69 | 19.00 | 12.80 | 18.56 | 15.99 | 21.34 | 16.40 | 18.91 | 10.51 | 9.97 | 15.71 | 13.13 | 12.53 | 24.02 | 17.87 | 14.32 | 10.09 | 15.20 |
| 60-70 | 26.03 | 24.59 | 23.59 | 27.68 | 25.30 | 30.60 | 24.83 | 28.59 | 22.86 | 19.97 | 25.06 | 24.05 | 22.70 | 31.65 | 28.49 | 24.22 | 21.71 | 23.92 |
| 70- 80 | 27.60 | 23.11 | 28.02 | 26.59 | 26.29 | 24.23 | 26.67 | 26.50 | 32.34 | 27.44 | 27.22 | 27.67 | 29.50 | 21.17 | 28.01 | 29.14 | 30.84 | 26.44 |
| 80- 90 | 15.22 | 13.85 | 20.89 | 13.29 | 18.41 | 7.71 | 16.49 | 11.43 | 23.74 | 24.23 | 16.95 | 19.42 | 21.49 | 5.99 | 12.60 | 18.39 | 23.01 | 18.90 |
| 90- 100 | 3.32 | 3.64 | 7.74 | 2.71 | 5.49 | 1.59 | 4.57 | 1.95 | 5.23 | 12.68 | 5.28 | 8.94 | 7.16 | 0.95 | 2.35 | 4.97 | 8.33 | 6.32 |

**Table S4.** Deciduousness in Chhtota Nagur ecoregion in different years (2001 to 2018).

| Class / year | 2001 | 2002 | 2003 | 2004 | 2005 | 2006 | 2007 | 2008 | 2009 | 2010 | 2011 | 2012 | 2013 | 2014 | 2015 | 2016 | 2017 | 2018 |
| --- | --- | --- | --- | --- | --- | --- | --- | --- | --- | --- | --- | --- | --- | --- | --- | --- | --- | --- |
| LD | 0.07 | 0.06 | 0.08 | 0.05 | 0.04 | 0.06 | 0.08 | 0.07 | 0.05 | 0.06 | 0.07 | 0.09 | 0.08 | 0.11 | 0.12 | 0.15 | 0.14 | 0.18 |
| MD | 4.89 | 6.92 | 3.22 | 5.82 | 3.98 | 8.21 | 10.53 | 5.09 | 2.64 | 2.78 | 3.40 | 2.16 | 4.70 | 13.02 | 5.52 | 4.72 | 1.43 | 5.69 |
| HD | 61.82 | 60.39 | 49.38 | 64.55 | 58.11 | 56.97 | 70.47 | 69.70 | 47.83 | 43.27 | 41.34 | 51.50 | 50.60 | 69.63 | 62.68 | 46.76 | 44.27 | 60.00 |
| VHD | 33.22 | 32.64 | 47.33 | 29.58 | 37.88 | 34.75 | 18.91 | 25.14 | 49.48 | 53.89 | 55.19 | 46.24 | 44.61 | 17.23 | 31.68 | 48.37 | 54.16 | 34.14 |

(Note: Blue is a wet year and Red is a dry year).

**Table S5.** Deciduousness in Narmada Valley ecoregion in different years (2001 to 2018).

| Class / year | 2001 | 2002 | 2003 | 2004 | 2005 | 2006 | 2007 | 2008 | 2009 | 2010 | 2011 | 2012 | 2013 | 2014 | 2015 | 2016 | 2017 | 2018 |
| --- | --- | --- | --- | --- | --- | --- | --- | --- | --- | --- | --- | --- | --- | --- | --- | --- | --- | --- |
| LD | 0.06 | 0.11 | 0.14 | 0.03 | 0.04 | 0.11 | 0.12 | 0.09 | 0.08 | 0.11 | 0.14 | 0.16 | 0.15 | 0.22 | 0.13 | 0.18 | 0.08 | 0.16 |
| MD | 7.97 | 11.78 | 6.87 | 6.01 | 4.24 | 8.80 | 6.73 | 4.20 | 2.96 | 4.18 | 5.21 | 6.51 | 2.97 | 9.60 | 6.33 | 4.77 | 2.46 | 5.35 |
| HD | 55.88 | 58.09 | 51.21 | 55.24 | 41.42 | 63.55 | 49.87 | 53.46 | 40.54 | 43.32 | 45.65 | 59.28 | 38.19 | 68.32 | 55.50 | 47.18 | 40.34 | 44.97 |
| VHD | 36.09 | 30.01 | 41.78 | 38.72 | 54.29 | 27.54 | 43.28 | 42.25 | 56.42 | 52.4 | 49 | 34.05 | 58.7 | 21.86 | 38.03 | 47.85 | 57.12 | 49.51 |

(Note: Blue is a wet year and Red is a dry year).

**Table S6**. Deciduousness in Eastern Highlands ecoregion in different years (2001 to 2018).

| Class / year | 2001 | 2002 | 2003 | 2004 | 2005 | 2006 | 2007 | 2008 | 2009 | 2010 | 2011 | 2012 | 2013 | 2014 | 2015 | 2016 | 2017 | 2018 |
| --- | --- | --- | --- | --- | --- | --- | --- | --- | --- | --- | --- | --- | --- | --- | --- | --- | --- | --- |
| LD | 0.05 | 0.09 | 0.03 | 0.03 | 0.07 | 0.06 | 0.07 | 0.16 | 0.02 | 0.02 | 0.11 | 0.02 | 0.10 | 0.11 | 0.05 | 0.11 | 0.04 | 0.05 |
| MD | 4.41 | 9.05 | 1.97 | 5.27 | 4.41 | 10.68 | 5.26 | 10.08 | 1.61 | 1.62 | 7.02 | 2.47 | 4.01 | 12.22 | 6.21 | 4.78 | 1.92 | 5.25 |
| HD | 51.59 | 53.62 | 41.14 | 58.90 | 53.29 | 67.42 | 47.78 | 60.01 | 40.03 | 34.29 | 55.13 | 40.61 | 47.03 | 68.43 | 59.18 | 49.50 | 39.75 | 49.07 |
| VHD | 43.96 | 37.23 | 56.85 | 35.8 | 42.22 | 21.83 | 46.89 | 29.75 | 58.33 | 64.07 | 37.74 | 56.9 | 48.86 | 19.24 | 34.55 | 45.6 | 58.29 | 45.64 |

(Note: Blue is a wet year and Red is a dry year).

**Table S7**. Deciduousness in Northern Dry Deciduousness ecoregion in different years (2001 to 2018).

| Class / year | 2001 | 2002 | 2003 | 2004 | 2005 | 2006 | 2007 | 2008 | 2009 | 2010 | 2011 | 2012 | 2013 | 2014 | 2015 | 2016 | 2017 | 2018 |
| --- | --- | --- | --- | --- | --- | --- | --- | --- | --- | --- | --- | --- | --- | --- | --- | --- | --- | --- |
| LD | 0.16 | 0.10 | 0.12 | 0.10 | 0.14 | 0.12 | 0.14 | 0.17 | 0.13 | 0.12 | 0.12 | 0.11 | 0.14 | 0.11 | 0.10 | 0.09 | 0.08 | 0.07 |
| MD | 3.45 | 5.50 | 1.31 | 3.63 | 4.30 | 9.33 | 3.55 | 6.43 | 1.56 | 0.83 | 5.66 | 1.05 | 2.52 | 6.94 | 3.75 | 3.81 | 1.02 | 3.35 |
| HD | 45.18 | 48.10 | 34.89 | 55.92 | 51.07 | 52.25 | 44.95 | 54.27 | 39.28 | 29.78 | 49.29 | 30.79 | 41.46 | 52.49 | 51.86 | 38.13 | 31.39 | 40.57 |
| VHD | 51.21 | 46.32 | 63.69 | 40.35 | 44.49 | 38.3 | 51.36 | 39.13 | 59.03 | 69.28 | 44.93 | 68.05 | 55.88 | 40.45 | 44.29 | 57.96 | 67.51 | 56.01 |

(Note: Blue is a wet year and Red is a dry year).

**Table S8.** Percentage Rainfall Anomaly (PRA) in Chhtota Nagur ecoregion.

| Class / Year | 2001 | 2002 | 2003 | 2004 | 2005 | 2006 | 2007 | 2008 | 2009 | 2010 | 2011 | 2012 | 2013 | 2014 | 2015 | 2016 | 2017 | 2018 |
| --- | --- | --- | --- | --- | --- | --- | --- | --- | --- | --- | --- | --- | --- | --- | --- | --- | --- | --- |
| < -25 | 0.00 | 1.25 | 0.00 | 0.00 | 9.54 | 0.00 | 0.00 | 0.00 | 15.91 | 32.30 | 0.00 | 0.00 | 0.00 | 0.00 | 0.18 | 0.00 | 0.00 | 3.46 |
| -25 - -20 | 0.00 | 5.18 | 0.00 | 0.00 | 27.00 | 0.06 | 0.00 | 0.00 | 9.65 | 39.51 | 0.00 | 0.00 | 0.00 | 0.00 | 4.53 | 0.12 | 0.00 | 1.67 |
| -20 - -10 | 1.43 | 46.01 | 2.32 | 22.35 | 34.68 | 2.80 | 1.49 | 0.00 | 40.41 | 27.83 | 1.43 | 9.77 | 1.61 | 32.24 | 17.88 | 6.44 | 2.80 | 12.75 |
| -10 - 0 | 52.86 | 40.70 | 45.89 | 56.26 | 19.01 | 16.09 | 12.04 | 4.29 | 33.19 | 0.36 | 7.27 | 38.56 | 18.41 | 63.11 | 61.80 | 19.25 | 22.88 | 59.06 |
| 0 - 10 | 38.20 | 4.95 | 29.32 | 17.64 | 8.58 | 27.00 | 18.95 | 31.23 | 0.83 | 0.00 | 35.82 | 26.10 | 33.08 | 4.65 | 14.78 | 18.95 | 24.14 | 20.74 |
| 10 - -20 | 7.51 | 1.91 | 18.30 | 3.75 | 1.19 | 48.81 | 48.33 | 57.27 | 0.00 | 0.00 | 51.55 | 23.60 | 37.43 | 0.00 | 0.83 | 9.48 | 21.69 | 2.32 |
| 20 - 25 | 0.00 | 0.00 | 4.17 | 0.00 | 0.00 | 5.01 | 14.18 | 5.13 | 0.00 | 0.00 | 3.81 | 1.97 | 7.03 | 0.00 | 0.00 | 45.77 | 9.36 | 0.00 |
| > 25 | 0.00 | 0.00 | 0.00 | 0.00 | 0.00 | 0.24 | 5.01 | 2.09 | 0.00 | 0.00 | 0.12 | 0.00 | 2.44 | 0.00 | 0.00 | 0.00 | 19.13 | 0.00 |
| Positive PRA | **45.71** | **6.85** | **51.79** | **21.39** | **9.77** | **81.05** | **86.47** | **95.71** | **0.83** | **0.00** | **91.30** | **51.67** | **79.98** | **4.65** | **15.61** | **74.20** | **74.31** | **23.06** |
| Negative PRA | **54.29** | **93.15** | **48.21** | **78.61** | **90.23** | **18.95** | **13.53** | **4.29** | **99.17** | **100.00** | **8.70** | **48.33** | **20.02** | **95.35** | **84.39** | **25.80** | **25.69** | **76.94** |

(Note: **If the total Positive PRA area is > 75% then that year is considered as wet year**, and **if the total Negative PRA area is >75% then that year is considered as dry year**).

**Table S9.** Percentage Rainfall Anomaly in Narmada Valley ecoregion.

| Class / Year | 2001 | 2002 | 2003 | 2004 | 2005 | 2006 | 2007 | 2008 | 2009 | 2010 | 2011 | 2012 | 2013 | 2014 | 2015 | 2016 | 2017 | 2018 |
| --- | --- | --- | --- | --- | --- | --- | --- | --- | --- | --- | --- | --- | --- | --- | --- | --- | --- | --- |
| < -25 | 20.38 | 10.62 | 1.06 | 6.16 | 0.00 | 0.00 | 15.09 | 0.08 | 21.32 | 0.11 | 0.00 | 0.91 | 0.00 | 0.15 | 33.88 | 0.00 | 0.08 | 1.51 |
| -25 - -20 | 17.28 | 7.30 | 3.29 | 22.34 | 1.25 | 0.00 | 11.46 | 10.51 | 12.14 | 2.87 | 0.00 | 1.17 | 0.00 | 13.42 | 18.56 | 0.00 | 13.91 | 3.02 |
| -20 - -10 | 9.87 | 43.21 | 12.21 | 45.94 | 6.20 | 10.59 | 25.78 | 30.62 | 27.86 | 16.64 | 3.40 | 1.55 | 0.08 | 64.61 | 12.14 | 0.49 | 54.29 | 15.99 |
| -10 - 0 | 16.33 | 37.88 | 16.64 | 20.00 | 14.48 | 29.45 | 20.42 | 24.88 | 32.51 | 36.79 | 16.64 | 22.72 | 0.72 | 21.32 | 21.59 | 2.80 | 28.02 | 65.90 |
| 0 - 10 | 34.14 | 0.98 | 15.39 | 3.29 | 29.91 | 10.62 | 11.42 | 28.73 | 5.33 | 14.63 | 42.38 | 38.37 | 1.93 | 0.49 | 12.33 | 7.26 | 3.36 | 13.57 |
| 10 - -20 | 2.00 | 0.00 | 44.54 | 1.51 | 19.70 | 3.74 | 8.02 | 5.14 | 0.72 | 16.98 | 36.14 | 22.61 | 4.54 | 0.00 | 1.51 | 14.10 | 0.34 | 0.00 |
| 20 - 25 | 0.00 | 0.00 | 5.75 | 0.45 | 11.49 | 4.39 | 5.18 | 0.04 | 0.04 | 3.14 | 1.06 | 10.40 | 7.94 | 0.00 | 0.00 | 10.93 | 0.00 | 0.00 |
| > 25 | 0.00 | 0.00 | 1.13 | 0.30 | 16.98 | 41.21 | 2.65 | 0.00 | 0.08 | 8.85 | 0.38 | 2.27 | 84.80 | 0.00 | 0.00 | 64.42 | 0.00 | 0.00 |
| Positive PRA | **36.14** | **0.98** | **66.81** | **5.55** | **78.08** | **59.96** | **27.27** | **33.91** | **6.17** | **43.6** | **79.96** | **73.65** | **99.21** | **0.49** | **13.84** | **96.71** | **3.7** | **13.57** |
| Negative PRA | **63.86** | **99.01** | **33.2** | **94.44** | **21.93** | **40.04** | **72.75** | **66.09** | **93.83** | **56.41** | **20.04** | **26.35** | **0.8** | **99.5** | **86.17** | **3.29** | **96.3** | **86.42** |

(Note: **If the total Positive PRA area is > 75% then that year is considered as wet year**, and **if the total Negative PRA area is >75% then that year is considered as dry year**).

**Table S10**. Percentage Rainfall Anomaly in Eastern Highlands ecoregion.

| Class / Year | 2001 | 2002 | 2003 | 2004 | 2005 | 2006 | 2007 | 2008 | 2009 | 2010 | 2011 | 2012 | 2013 | 2014 | 2015 | 2016 | 2017 | 2018 |
| --- | --- | --- | --- | --- | --- | --- | --- | --- | --- | --- | --- | --- | --- | --- | --- | --- | --- | --- |
| < -25 | 2.97 | 25.87 | 0.00 | 3.67 | 0.15 | 0.00 | 0.00 | 0.00 | 0.00 | 7.76 | 1.44 | 0.00 | 0.00 | 0.00 | 4.12 | 0.00 | 0.27 | 0.00 |
| -25 - -20 | 1.21 | 27.76 | 0.00 | 3.08 | 1.49 | 0.85 | 0.00 | 0.12 | 0.50 | 10.73 | 9.82 | 0.38 | 0.00 | 0.80 | 11.77 | 0.05 | 6.34 | 0.00 |
| -20 - -10 | 3.98 | 42.75 | 0.80 | 35.28 | 12.59 | 13.65 | 0.99 | 3.49 | 4.68 | 13.10 | 16.08 | 5.69 | 0.00 | 17.63 | 49.72 | 5.76 | 27.88 | 8.82 |
| -10 - 0 | 26.28 | 3.62 | 13.67 | 54.25 | 16.66 | 19.24 | 12.28 | 44.40 | 34.79 | 13.67 | 15.63 | 15.02 | 2.14 | 62.94 | 33.74 | 34.60 | 25.15 | 38.85 |
| 0 - 10 | 39.76 | 0.00 | 32.60 | 3.72 | 34.80 | 36.85 | 58.89 | 30.07 | 46.51 | 14.80 | 31.20 | 43.57 | 30.26 | 18.59 | 0.65 | 39.72 | 26.94 | 45.93 |
| 10 - -20 | 25.61 | 0.00 | 37.60 | 0.00 | 21.63 | 27.92 | 20.86 | 18.62 | 11.86 | 17.09 | 25.76 | 27.81 | 33.37 | 0.03 | 0.00 | 14.92 | 11.52 | 6.41 |
| 20 - 25 | 0.17 | 0.00 | 12.80 | 0.00 | 5.57 | 1.45 | 4.20 | 3.04 | 1.67 | 5.71 | 0.07 | 4.80 | 10.39 | 0.00 | 0.00 | 3.19 | 1.62 | 0.00 |
| > 25 | 0.02 | 0.00 | 2.53 | 0.00 | 7.11 | 0.03 | 2.77 | 0.26 | 0.00 | 17.15 | 0.00 | 2.73 | 23.85 | 0.00 | 0.00 | 1.76 | 0.27 | 0.00 |
| Positive PRA | **65.56** | **0** | **85.53** | **3.72** | **69.11** | **66.25** | **86.72** | **51.99** | **60.04** | **54.75** | **57.03** | **78.91** | **97.87** | **18.62** | **0.65** | **59.59** | **40.35** | **52.34** |
| Negative PRA | **34.44** | **100** | **14.47** | **96.28** | **30.89** | **33.74** | **13.27** | **48.01** | **39.97** | **45.26** | **42.97** | **21.09** | **2.14** | **81.37** | **99.35** | **40.41** | **59.64** | **47.67** |

(Note: **If the total Positive PRA area is > 75% then that year is considered as wet year**, and **if the total Negative PRA area is >75% then that year is considered as dry year**).

**Table S11.** Percentage Rainfall Anomaly in Northern Dry Deciduous ecoregion.

| Class/Year | 2001 | 2002 | 2003 | 2004 | 2005 | 2006 | 2007 | 2008 | 2009 | 2010 | 2011 | 2012 | 2013 | 2014 | 2015 | 2016 | 2017 | 2018 |
| --- | --- | --- | --- | --- | --- | --- | --- | --- | --- | --- | --- | --- | --- | --- | --- | --- | --- | --- |
| < -25 | 0.00 | 32.44 | 0.00 | 0.00 | 0.00 | 0.00 | 0.00 | 0.00 | 0.00 | 16.43 | 0.00 | 0.00 | 0.00 | 0.00 | 0.00 | 0.00 | 0.00 | 0.00 |
| -25 - -20 | 0.00 | 43.22 | 0.00 | 0.00 | 0.00 | 0.00 | 0.00 | 0.00 | 3.08 | 10.88 | 4.41 | 0.00 | 0.00 | 0.00 | 3.49 | 1.75 | 0.00 | 0.00 |
| -20 - -10 | 0.00 | 24.33 | 0.00 | 18.79 | 9.24 | 0.00 | 0.00 | 0.00 | 23.61 | 20.53 | 21.25 | 8.01 | 0.00 | 2.77 | 60.88 | 35.63 | 5.03 | 0.00 |
| -10 - 0 | 12.83 | 0.00 | 20.02 | 73.10 | 30.08 | 9.14 | 4.41 | 5.65 | 66.53 | 22.18 | 24.64 | 10.47 | 9.86 | 72.59 | 35.63 | 60.88 | 52.05 | 24.74 |
| 0 - 10 | 52.77 | 0.00 | 47.95 | 8.11 | 48.97 | 34.29 | 55.13 | 20.33 | 6.78 | 11.81 | 18.17 | 34.91 | 66.22 | 24.44 | 0.00 | 1.75 | 42.30 | 57.49 |
| 10 - -20 | 34.39 | 0.00 | 31.42 | 0.00 | 11.70 | 55.85 | 32.85 | 61.70 | 0.00 | 2.87 | 30.39 | 36.55 | 22.07 | 0.21 | 0.00 | 0.00 | 0.62 | 17.76 |
| 20 - 25 | 0.00 | 0.00 | 0.62 | 0.00 | 0.00 | 0.72 | 7.60 | 8.73 | 0.00 | 2.67 | 1.13 | 9.65 | 1.64 | 0.00 | 0.00 | 0.00 | 0.00 | 0.00 |
| > 25 | 0.00 | 0.00 | 0.00 | 0.00 | 0.00 | 0.00 | 0.00 | 3.59 | 0.00 | 12.63 | 0.00 | 0.41 | 0.21 | 0.00 | 0.00 | 0.00 | 0.00 | 0.00 |
| Positive PRA | **87.16** | **0** | **79.99** | **8.11** | **60.67** | **90.86** | **95.58** | **94.35** | **6.78** | **29.98** | **49.69** | **81.52** | **90.14** | **24.65** | **0** | **1.75** | **42.92** | **75.25** |
| Negative PRA | **12.83** | **99.99** | **20.02** | **91.89** | **39.32** | **9.14** | **4.41** | **5.65** | **93.22** | **70.02** | **50.30** | **18.48** | **9.86** | **75.36** | **100** | **98.26** | **57.08** | **24.74** |

(Note: **If the total Positive PRA area is > 75% then that year is considered as wet year**, and **if the total Negative PRA area is >75% then that year is considered as dry year**).

**Table S12.** Deciduousness distribution (in % area) under different elevation zones in the study area.

| **Elevation Zones** | **LD** | **MD** | **HD** | **VHD** |
| --- | --- | --- | --- | --- |
| 1 to 100 m | 0.00 | 0.28 | 1.46 | 0.39 |
| 100 to 200 m | 0.01 | 0.67 | 4.21 | 2.34 |
| 200 to 300 m | 0.01 | 0.58 | 6.96 | 6.22 |
| 300 to 400 m | 0.01 | 0.81 | 10.42 | 8.25 |
| 400 to 500 m | 0.01 | 1.11 | 12.63 | 8.38 |
| 500 to 600 m | 0.00 | 0.41 | 8.54 | 6.10 |
| 600 to 700 m | 0.01 | 0.32 | 5.67 | 2.88 |
| 700 to 800 m | 0.05 | 0.36 | 3.56 | 1.41 |
| > 800 m | 0.08 | 0.60 | 4.39 | 0.88 |
| Total | **0.19** | **5.12** | **57.84** | **36.85** |
